# A unique cell wall synthetic response evoked by glucosamine determines pathogenicity-associated fungal cellular differentiation

**DOI:** 10.1371/journal.pgen.1009817

**Published:** 2021-10-08

**Authors:** Pengjie Hu, Hao Ding, Lan Shen, Guang-Jun He, Huimin Liu, Xiuyun Tian, Changyu Tao, Xiangzheng Bai, Jingnan Liang, Cheng Jin, Xinping Xu, Ence Yang, Linqi Wang

**Affiliations:** 1 State Key Laboratory of Mycology, Institute of Microbiology, Chinese Academy of Sciences, Beijing, China; 2 University of Chinese Academy of Sciences, Beijing, China; 3 University of Science and Technology of China (USTC), Hefei, China; 4 Department of Microbiology, School of Basic Medical Sciences, Peking University Health Science Center, Beijing, China; 5 Public Technology Service Center, Institute of Microbiology, Chinese Academy of Sciences, Beijing, China; 6 The First Affiliated Hospital of Nanchang University, Nanchang, China; Duke University Medical Center, UNITED STATES

## Abstract

The yeast-to-hypha transition is tightly associated with pathogenicity in many human pathogenic fungi, such as the model fungal pathogen *Cryptococcus neoformans*, which is responsible for approximately 180,000 deaths annually. In this pathogen, the yeast-to-hypha transition can be initiated by distinct stimuli: mating stimulation or glucosamine (GlcN), the monomer of cell wall chitosan. However, it remains poorly understood how the signal specificity for *Cryptococcus* morphological transition by disparate stimuli is ensured. Here, by integrating temporal expression signature analysis and phenome-based clustering evaluation, we demonstrate that GlcN specifically triggers a unique cellular response, which acts as a critical determinant underlying the activation of GlcN-induced filamentation (GIF). This cellular response is defined by an unusually hyperactive cell wall synthesis that is highly ATP-consuming. A novel cell surface protein Gis1 was identified as the indicator molecule for the GlcN-induced cell wall response. The Mpk1-directed cell wall pathway critically bridges global cell wall gene induction and intracellular ATP supply, ensuring the Gis1-dependent cell wall response and the stimulus specificity of GIF. We further reveal that the ability of Mpk1 to coordinate the cell wall response and GIF activation is conserved in different *Cryptococcus* pathogens. Phosphoproteomics-based profiling together with genetic and phenotypic analysis revealed that the Mpk1 kinase mediates the regulatory specificity of GIF through a coordinated downstream regulatory network centered on Skn7 and Crz1. Overall, our findings discover an unprecedented and conserved cell wall biosynthesis-dependent fungal differentiation commitment mechanism, which enables the signal specificity of pathogenicity-related dimorphism induced by GlcN in *Cryptococcus* pathogens.

## Author summary

Many human fungal pathogens can undergo dimorphic transition between yeast and hyphal forms in response to different external stimuli, and this morphological transition is generally and critically linked with their infections. In *Cryptococcus neoformans*, a model pathogenic fungus, the yeast-to-hypha transition can be elicited by mating stimulation or glucosamine (GlcN), the monomer of cell wall chitosan. Here, we show that GlcN specifically evokes a unique hyperactive cell wall synthetic response, which determines GlcN-induced filamentation (GIF) as a key commitment event. The Mpk1-directed cell wall signaling pathway as a core and conserved cascade connects the cell wall synthetic response and GIF activation in different *Cryptococcus* pathogens. Overall, the findings reveal a previously unrecognized function of GlcN in stimulating cell wall signaling and biosynthetic machinery, which enables a unique dimorphism commitment mechanism underlying the signal specificity of the mating-independent yeast-to-hypha transition in *Cryptococcus* pathogens.

## Introduction

The life cycle of every fungus recognized to date involves multiple morphotypes. The transition between different morphotypes confers genetically identical fungal cells the distinct abilities in responding to various external stimuli, optimizing fungal survival under adverse environmental conditions or during infections. The yeast-to-hypha transition (also named dimorphic transition) represents the most-studied morphological switch in fungi, since many dimorphic fungi are important plant and animal pathogens and dimorphism shapes their pathogenicity [1–3]. Despite this, the molecular mechanisms underlying fungal dimorphism are still far from fully understood, even in model fungal species. This is largely due to the regulatory complexity involved in the stimulus specificity of fungal dimorphism by diverse external cues from natural niches or hosts.

*Cryptococcus neoformans* is a model basidiomycete and an important environmental human fungal pathogen worldwide, which causes an estimated 180,000 death annually and can undergo pathogenicity-associated dimorphic transition [4–11]. In this pathogen, yeast morphology is essential for its infection, whereas hyphae are rarely observed in the clinical setting [10,11]. Notably, hyper-activation of hyphal formation by overexpressing Znf2, the master regulator of filamentation, not only abolished cryptococcal virulence but also induced efficient host immune protection against fatal yeast infection [10,11]. The inverse relationship between hyphae and pathogenicity in *C*. *neoformans* is similar to what have been reported in other environmental fungal pathogens like *Histoplasma capsulatum*, *Blastomyces dermatitidis*, and *Penicillium marneffei*, suggesting that the anti-virulence feature of hyphae might be conserved in different environmental pathogens [12–17]. Besides its involvement in pathogenicity, dimorphism also offers flexibility and resilience for *C*. *neoformans* in competitive environmental niches. The hyphal morphotype has been shown to help prevent engulfment by soil amoeba *Acanthamoeba castellanii*, the natural predator of *C*. *neoformans*, and confer an ecological benefit by promoting foraging for nutrients and mating partners [18,19]. The yeast-to-hypha dimorphic transition therefore serves as an important adaptation strategy of *C*. *neoformans* that contributes to fitness in natural niches and in interactions with different hosts.

**Fig 1:**
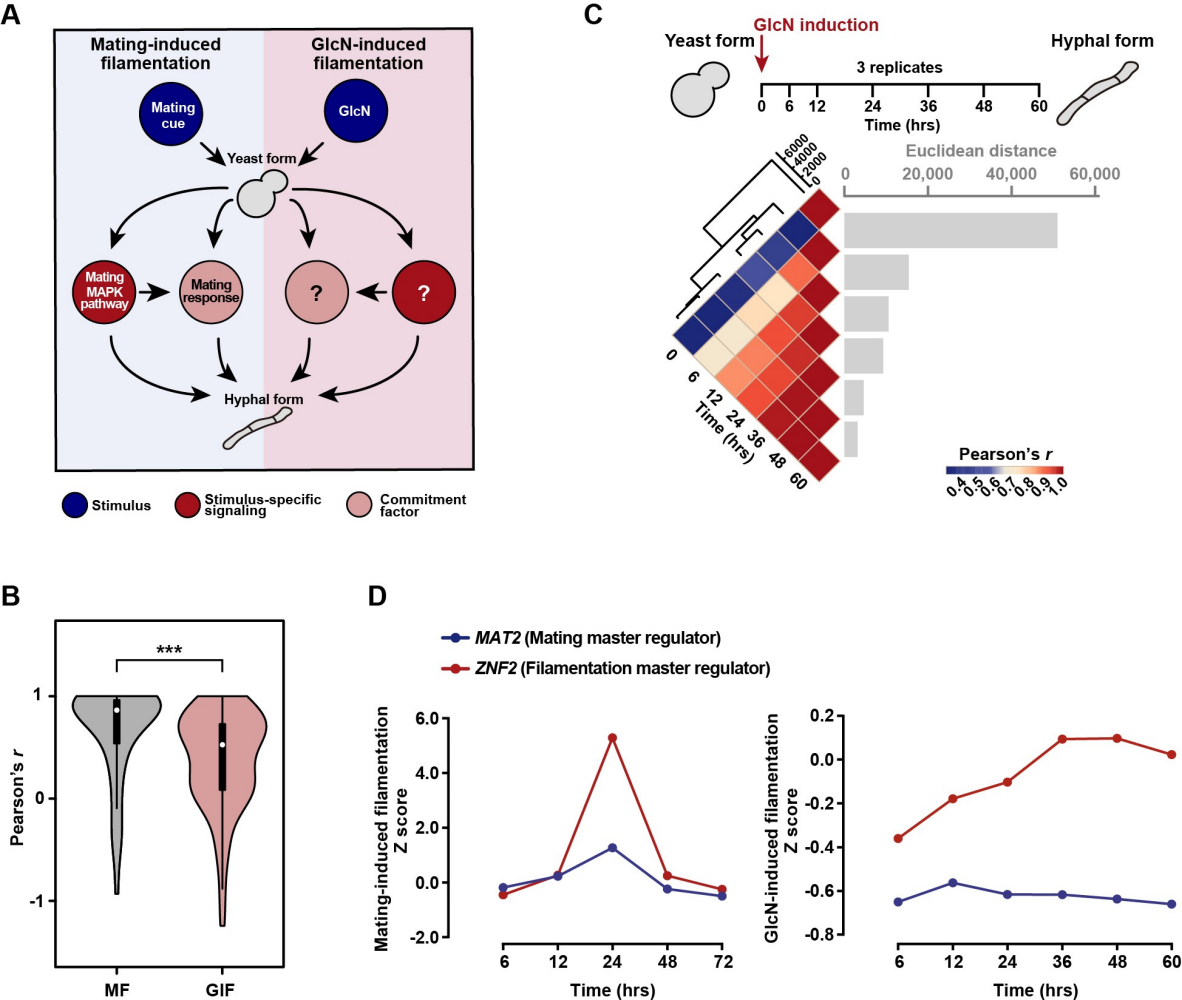
The mating MAPK signaling-directed mating response is not required for the activation of GIF. (A) Diagram depicting the yeast-to-hypha dimorphic transition induced by a mating stimulation and GlcN in *C*. *neoformans*. The mating MAPK cascade and mating response represent the central signaling pathway and cellular determinant underlying mating-induced filamentation. The commitment factor for GIF has yet to be revealed in *C*. *neoformans*. (B) Pearson correlation analysis for correlation of gene-expression dynamics between the mating MAPK genes during mating-induced filamentation and GlcN-induced filamentation. The cryptococcal gene expression data under mating-inducing conditions were derived from our previous research [20]. ***P < 0.001, Wilcoxon signed-rank test. MF, mating filamentation. (C) Pairwise correlation of normalized TPM between RNA-seq samples (bottom panel) obtained at seven time points after GlcN induction (upper panel). The Pearson correlation coefficient was calculated using the R package and ranges from a weak correlation (dark blue) to a perfect correlation (red). Euclidean distance was calculated to reflect the difference in gene expression between the two samples (bottom panel). Hierarchical clustering revealed major divisions between samples, and the distance (y-axis 0, 2000, 4000, 6000) indicates how closely the samples are related, with smaller values indicating smaller distances and stronger correlations. (D) Line charts of Z-scores for the expression levels of *MAT2* and *ZNF2* at various time points under mating-inducing condition or under GlcN-inducing condition.

Dimorphism in *C*. *neoformans* has been well recognized to be associated with mating, which can be efficiently initiated by coculture of cells of opposite mating types (*MAT***a** and *MAT*α) on mating-inducing media, such as MS or V8 juice agar. Prior to sexual filamentation, mating stimulation elicits a highly synchronized induction of mating-related genes at the early sexual stage, especially the genes encoding the components of the mating MAPK (mitogen-activated protein kinase) pathway, which is the pivotal mating signaling cascade [10,20]. This mating response represents a key commitment factor underlying mating filamentation [21]. Mat2, the output regulator of the mating MAPK cascade, mediates the mating response, further triggering the expression of Znf2 to initiate hyphal growth [21–23] (Fig 1A). Besides mating stimulation, it has been shown that the yeast-to-hypha transition can also be induced by glucosamine (GlcN), which is the monomeric unit of fungal cell wall chitosan [24] (Fig 1A). Intriguingly, many mating MAPK components, including Mat2 and the MAP kinase Cpk1, appear to be largely dispensable for GlcN-induced filamentation (GIF) [24], implicating that GIF does not require the mating response for its commitment. However, the commitment mechanism for GlcN-induced dimorphism in *C*. *neoformans* has yet to be addressed.

In this study, by monitoring expression dynamics throughout different stages after GlcN induction, we demonstrate that the mating MAPK pathway-directed mating response does not occur during GIF. Instead, the commitment to GIF requires a special cell wall synthetic response that relies on a high level of ATP supply and the GlcN-responsive cell surface protein Gis1. We show that the Mpk1-directed cell wall pathway serves as the central cascade that governs the cell wall response and determines the signal specificity of GIF in different *Cryptococcus* pathogenic species. Phosphoproteomics-based profiling together with genetic and phenotypic assays explored the regulators Skn7 and Crz1 as the central components downstream of the kinase Mpk1 mediates the regulatory specificity of GIF. Overall, our findings reveal a previously unrecognized role of GlcN, the monomer of cell wall chitosan, in stimulating cell wall signaling and biosynthesis, which enables a unique and conserved fungal differentiation commitment mechanism in different *Cryptococcus* pathogens.

## Results

### The mating MAPK signaling-directed mating response does not take place during GlcN-induced filamentation

Previous studies have revealed a concomitant induction of multiple mating MAPK genes upon mating stimulation [20,21] (Fig 1B), which reflects a critical mating response that is required for the subsequent hyphal initiation. To determine whether this mating response also occurs during GIF, we sought to examine the gene-expression dynamics of the mating MAPK genes at various time points after GlcN induction. To achieve this, high-coverage RNA-sequencing analysis was performed to compare whole-genome expression at 7 time points (0 hr, 6 hrs, 12 hrs, 24 hrs, 36 hrs, 48 hrs and 60 hrs) after transferring yeast cells onto GIF-inducing medium (Fig 1C). No later time point was tested because hyphae can be detected at 60 hrs after induction by GlcN and the genes affecting commitment to GIF should exhibit the transcriptional response earlier (S1A Fig). We obtained a total of 8268 unigenes from 7 time points, which included 6962 genes predicted to encode proteins, covering 99.81% of protein-coding genes in *C*. *neoformans* H99. Among them, 3950 genes that exhibited significantly differential expression (|log_2_(fold change)| > 1, *P*_*adj*_ < 0.01, transcripts per million mapped (TPM) > 10) at least at one time point tested were identified (S1 Table). Pairwise correlation analysis indicated highly dynamic gene expression profiling until 36 hrs after GlcN induction (Fig 1C and S1 Table). We found that the gene-expression dynamics of the key mating response genes were poorly correlated during GIF (Fig 1B). This is in sharp contrast to the highly synchronous induction of these genes during mating filamentation (Fig 1B), which was based on analysis of previous time-series transcriptomic data of the yeast-to-hypha transition induced by mating cue [20]. In addition, we did not detect the evident induction of *MAT2*, the master activator for the cryptococcal mating response [21], during GIF (Fig 1D). Rather, *MAT2* was expressed at a basal level throughout all time points tested after GlcN induction, consistent with an earlier study showing an unnecessary role of Mat2 in GIF [24] (Fig 1D). These results suggest that the cellular response to mating stimulation mediated by Mat2 may not take place during GIF. In support of this conclusion, quantitative α-**a** cell fusion assays indicated that GlcN strongly inhibited α-**a** mating, which depends on the proper mating response directed by Mat2 [21], confirming that GIF is mating-independent (S1B Fig). Unlike *MAT2*, the dramatically induced transcription of *ZNF2* was detected during both GIF and mating filamentation (Fig 1D), echoing the role of Znf2 as the filamentation master regulator in both GIF and mating-dependent dimorphism [10,21,24].

### GlcN evokes a pre-filamentation cell wall synthetic response that underlies GIF initiation

**Fig 2:**
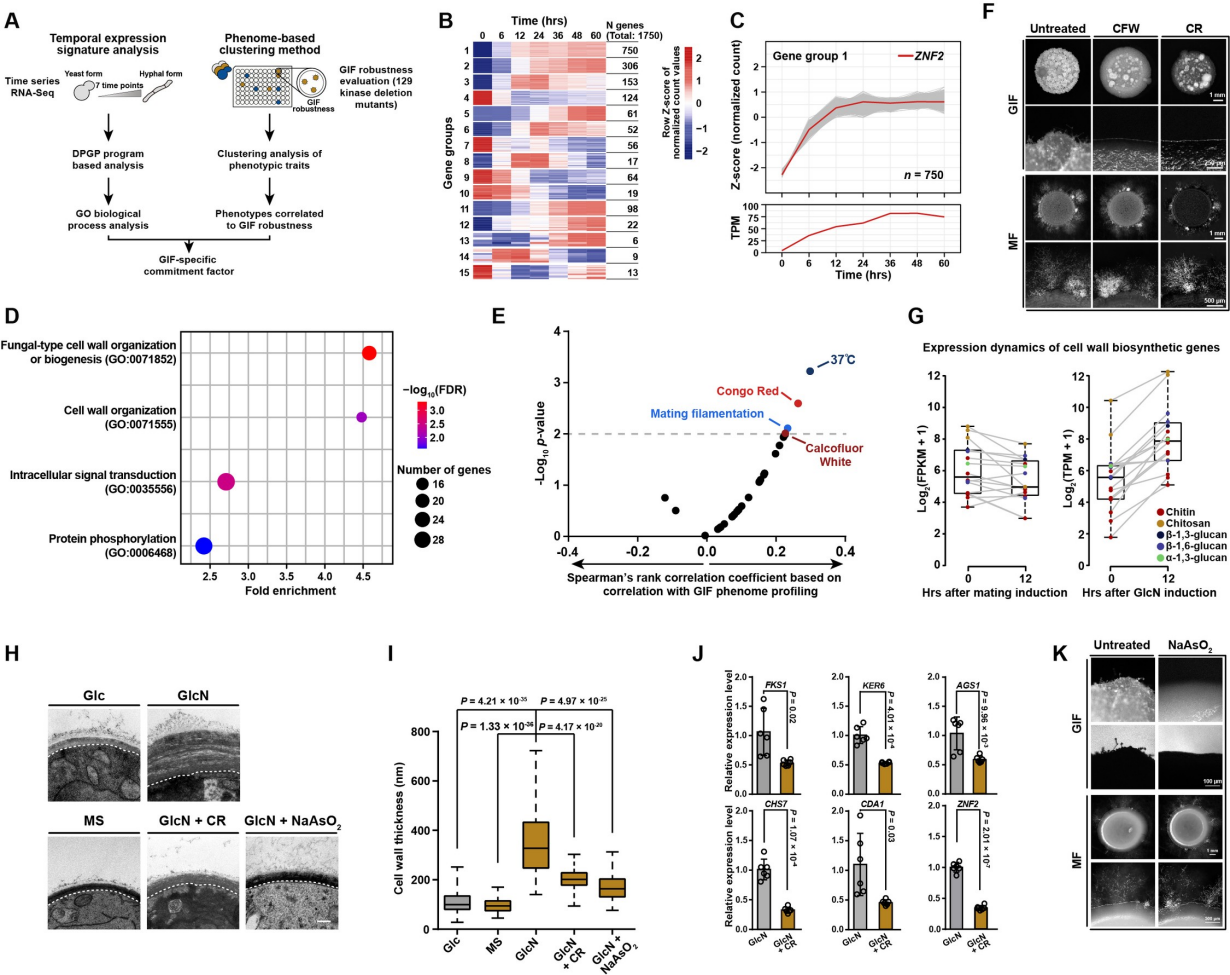
Integrating two parallel systematic approaches revealed that GlcN induces a unique cell wall synthetic response underlying the initiation of GIF. (A) Schematic diagram depicting two parallel systematic approaches applied to identifying the commitment factor underlying GIF. (B) Transcriptional profiling of 15 gene groups identified according to the DP_GP_cluster-based analysis of the time-series RNA-seq data that targeted different stages after GlcN induction. The color bar indicates Z-score of normalized counts. (C) Expression patterns of genes belonging to group 1 based on their temporal expression signature during the GlcN-induced yeast-to-hypha transition. The temporal expression of *ZNF2* is shown with a red line based on normalized count (Upper) and TPM (Bottom). (D) Gene ontology biological process enrichment analysis of genes in group 1. FDR, False discovery rate; GO, Gene Ontology. (E) Functional correlation between the GIF phenotype and various phenotypic traits. Correlations based on Spearman’s rank correlation analysis were calculated between GIF robustness data generated in this study and the phenome data from a previous study [26]. The gray dashed line indicates *P* = 0.01. (F) Phenotypes of GIF and mating filamentation in the presence of CFW or CR. For GIF, the yeast cells were spotted on YPGlcN medium and YPGlcN medium supplemented with CFW or CR at subinhibitory concentrations and incubated in the dark for 7 days before they were photographed. For mating filamentation, a mixture of H99α and KN99**a** cells was cocultured on mating medium in the absence or presence of CFW or CR at subinhibitory concentrations. Filamentation was photographed after 14 days of incubation. MF, mating filamentation. (G) The induction of *Cryptococcus* published cell wall biosynthetic genes upon stimulation by mating cue or GlcN. The genes related to the biosynthesis of cell wall components are color coded, see inset legend. The boxplots represent the medians and interquartile ranges. The data for cryptococcal gene expression under the mating-inducing condition were derived from previous research. [20]. (H) TEM-based visualization of cell wall morphology of the H99 yeast cells after 48 hrs of incubation under different conditions. Glc: YP base medium plus 2% glucose; GlcN: YP base medium plus 2% glucosamine; GlcN + CR: YP base medium plus 2% glucosamine and 1% Congo red; GlcN + NaAsO_2_: YP base medium plus 2% glucosamine and 1 mM NaAsO_2_. Scale bar, 200 nm. (I) Cell wall thickness of the H99 yeast cells cultured under different conditions. The cells cultured in media containing GlcN showed a significantly thicker cell wall than those cultured in Glc (glucose) or MS (mating-inducing) media. GlcN-induced cell wall growth was substantially repressed by the addition of CR or NaAsO_2_. For each condition, 100 yeast cells were randomly selected and used to calculate the cell wall thickness. The boxplots represent the medians and interquartile ranges. Two-tailed Student’s *t*-test. (J) qRT-PCR showing the mRNA levels of various genes from the H99 cells cultured on YPGlcN in the absence or presence of 1% CR. Fks1, Ker6, Ags1, Chs7, and Cda1 are involved in the synthesis of the cell wall components β-1,3-glucan, β-1,6-glucan, α-1,3-glucan, chitin and chitsosan, respectively [46,47]. Data are presented as the mean ± SD of six independent experiments, two-tailed Student’s *t*-test. (K) Phenotypes of GIF and mating filamentation in the presence or absence of 1 mM NaAsO_2_.

The aforementioned results suggest that the mating response does not occur during GIF, which may require an unknown commitment factor for its initiation. To identify such factor, two parallel systematic approaches were applied (Fig 2A). Firstly, we employed Dirichlet process Gaussian process mixture model to analyze the time-series RNA-seq data of GIF for grouping genes according to their temporal expression patterns (Fig 2A). As a result, a total of 15 gene groups were identified (Fig 2B). We focused on the gene group (group 1) that included the coding gene of the filamentation-specific master regulator Znf2, which suggests that the genes in this group might be functionally related to filamentation (Fig 2C). GO enrichment analysis explored multiple GO terms enriched in this group, including the ones related to cell wall biogenesis/organization, intracellular signaling transduction, and protein phosphorylation, suggesting a potential complexity of molecular/cellular processes involved in GIF (Fig 2C). To further determine the underlying event of GIF, we used a phenome-based clustering method reported before [25,26]. We semiquantitatively assessed 129 kinase deletion mutants constructed by Yong-Sun Bahn’s group for the phenotypic trait related to GIF robustness [25] (S2 Fig and S2 Table). Previously, all these kinase mutants had been characterized for a range of other phenotypic traits under 30 distinct *in vitro* conditions [25]. The comprehensive data of the functional profiling of these kinase mutants made it possible to identify the phenotypes significantly correlated to GIF robustness (Spearman’s rank correlation analysis, *P* < 0.01) (Fig 2E). Using this method, GIF was found to be strongly correlated with four phenotypes, as shown in Fig 2E. These phenotypes included mating filamentation, suggesting that some kinases are shared in both mating filamentation and GIF. This idea is in agreement with previous studies indicating that some kinase components from the high-osmolarity glycerol (HOG) pathway negatively controlled both filamentation modes [24,25]. The other three phenotypes correlated with GIF were associated with cellular growth at host temperature (37°C) or under stress by the compounds Congo red (CR) and Calcofluor white (CFW) (Fig 2E). Notably, both CFW and CR are well known for their abilities to perturb cell wall synthesis [27]. We further proved that either CR or CFW can completely prevent GIF at a subinhibitory concentration (Fig 2F). In contrast, these cell wall-perturbing agents with the same concentration did not result in detectable change in mating filamentation (Fig 2F). This specific inhibition of GIF by the cell wall-perturbing compounds likely reflects that there may exist a cell wall-associated process, which is specifically required for the initiation of GIF. This idea was supported by the aforementioned GO analysis (Fig 2D), which showed that the top two GO terms enriched in the *ZNF2*-included gene group were both related to cell wall processes, including cell wall organization (GO:0071555) and fungal-type cell wall organization or biogenesis (GO:0071852). Time-series RNA-seq analysis further revealed that the biosynthetic genes for major cell wall components of *C*. *neoformans* were globally induced at the early stage of GIF (12 hrs after induction) (Figs 2G and S3A). Such induction was undetected during mating filamentation (Fig 2G), indicating that it is specific for GIF.

We next hypothesized that the global induction of cell wall biosynthetic genes may lead to an enhancement in cell wall growth. To address this hypothesis, transmission electron microscopy (TEM) analysis was performed to visualize the changes in cell wall morphology and architecture in response to GlcN (Figs 2H, 2I and S3B). Strikingly, in the presence of GlcN, the cells had a considerably thicker cell wall than those cultured under the filamentation-repressing (YPD medium) and mating-inducing (MS medium) conditions (Fig 2H and 2I). GlcN-stimulated cell walls were observed to be approximately three fold thicker than those of cells grown in YPD or mating media. Besides, the cell walls in a majority of the cell population contained multiple layers of architecture upon GlcN induction (S3B Fig), but such architecture was not observed in cells grown in YPD medium or under mating-inducing condition (Fig 2H). We further showed that the cell wall-interfering compound CR substantially suppressed the GlcN-induced cell wall thickening (Fig 2H and 2I). Consistently, qRT-PCR-based transcription assays indicated a significant decrease in the mRNA levels of the key genes involved in the biosynthesis of various components of the cryptococcal cell wall when CR and GlcN were both present (Fig 2J). These evidence suggest that GlcN, the monomer of cell wall chitosan, induces a hyperactive cell wall synthetic response at the early stage of GIF.

It has been well recognized that fungal cell wall synthesis is an energy-consuming process [28,29], and we therefore reasoned that the hyperactive cell wall synthesis in response to GlcN signal likely requires a high level of ATP supply. To test this idea, the cells were treated with arsenite, an inhibitor of ATP synthesis, under GIF-inducing or mating-inducing conditions. As illustrated in S3C Fig, the intracellular ATP content significantly reduced in the presence of arsenite at a subinhibitory concentration. The perturbation of ATP synthesis considerably inhibited the GlcN-induced cell wall growth and prevented GIF (Fig 2H and 2I). Unlike the strong inhibitory effect on GIF, treatment with arsenite appeared not to influence the mating-induced dimorphic transition (Figs 2K and S3C).

In fungi, cell wall biogenesis/remodeling processes are often coupled with the dimorphic transition [30,31], and the filamentation regulator co-regulates genes involved in cellular differentiation and cell wall process, enabling the coupling [30,31]. In such cases, cell wall activity reflects the morphogenesis-concomitant process rather than the underlying event. To corroborate that the cell wall synthetic response is not simply a morphogenesis-coupled process but the commitment event preceding and underlying the yeast-to-hypha transition induced by GlcN, we identified the regulon of the *Cryptococcus* filamentation-specific regulator Znf2 using RNA-seq-based transcriptomic analysis of the *znf2*Δ mutant under GIF-inducing condition (S3 Table). This analysis was performed at 12 hrs after stimulation by GlcN when many cell wall biogenesis genes already showed transcriptional responses to GlcN (Figs 2G and S3A). In the absence of Znf2, we did not detect any GlcN-induced cell wall-related gene that displayed significantly differential expression (|log_2_(fold change)| > 1, *P*_*adj*_ < 0.01) (S3D Fig and S3 Table). This finding suggests that the GlcN-induced cell wall response is not mediated by filamentation-specific activator Znf2 and is unlikely to be a filamentation-coupled event. Furthermore, the inhibition of the GlcN-stimulated cell wall response by CR led to the significantly attenuated expression of *ZNF2* (Fig 2J). These findings demonstrate that the cell wall synthetic response is upstream of, and required for, the activation of *ZNF2* during GIF.

We asked whether the cell wall response induced by GlcN and its requirement for GIF are unique to *C*. *neoformans*. To achieve this, we used TEM analysis to evaluate the change in cell wall structure in response to GlcN in the human fungal pathogen *Cryptococcus gattii*, which was diverged from *C*. *neoformans* more than 16 million years ago [32–34] (S3E Fig). We likewise observed an increased cell wall width upon GlcN induction in this fungus (S3E Fig). Importantly, CR-mediated perturbation of the cell wall response can also prevent GIF and inhibit GlcN-induced cell wall growth in this *Cryptococcus* pathogen (S3F Fig).

### A novel surface protein Gis1 can be used as an indicator of the pre-filamentation cell wall synthetic response induced by GlcN

**Fig 3:**
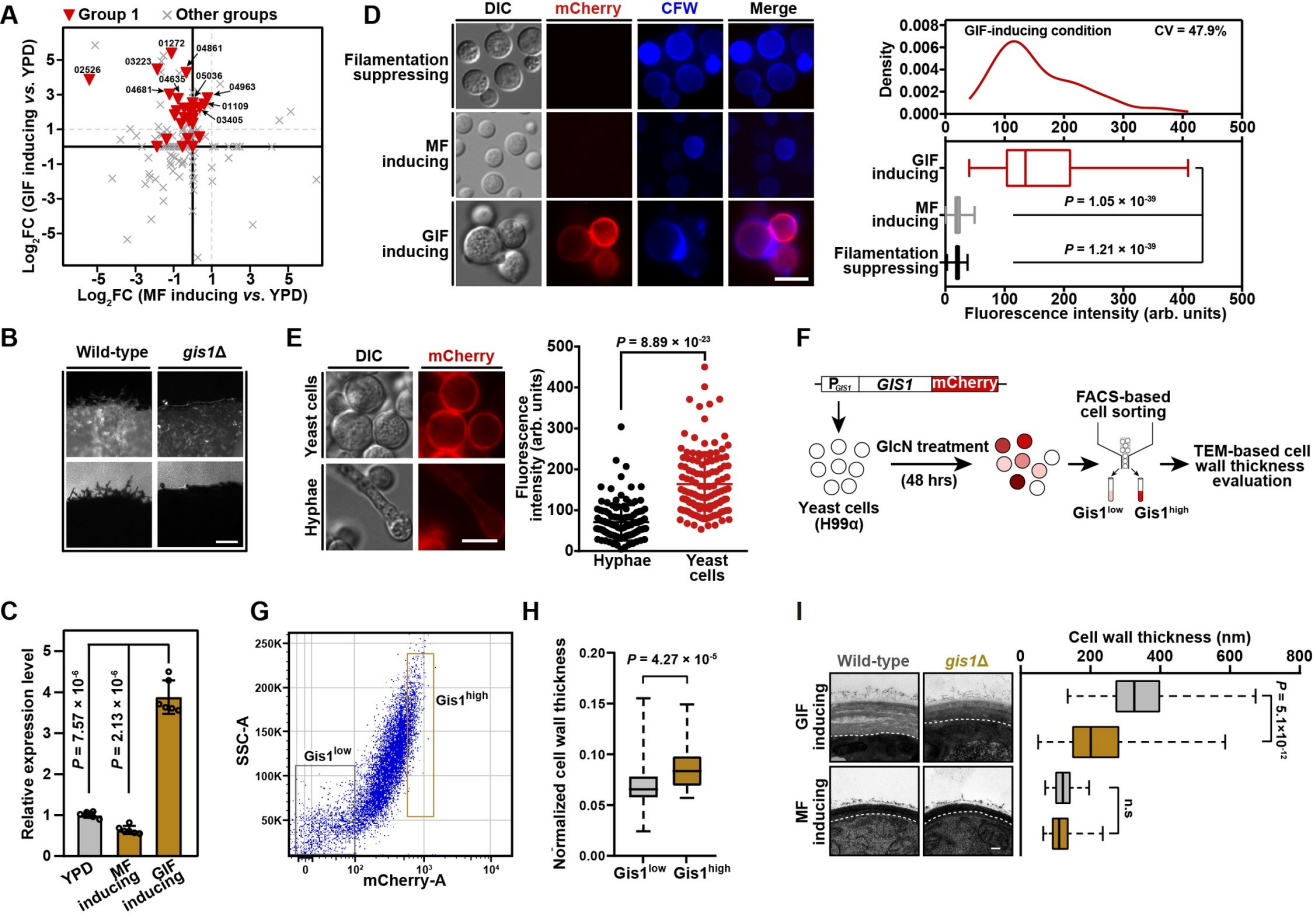
Gis1 as a GlcN-responsive surface protein is required for the pre-filamentation cell wall synthetic response during GIF. (A) Transcriptional response of predicted secretory/surface protein-coding genes to GlcN stimulation or mating cue. CNAG_‘number’ indicates the NCBI locus name of the selected genes that showed strong induction in response to GlcN stimulation (log_2_FC (GIF-inducing *vs*. YPD) > 1) but not mating cue (log_2_FC (MF-inducing *vs*. YPD) < 1). (B) Phenotype of GIF in the *gis1*Δ mutant. Cells of different strains were cultured on YPGlcN agar for 7 days. Scale bar, 100 μm. (C) qRT-PCR showing the mRNA levels of *GIS1* under filamentation-suppressing condition, GlcN-inducing condition or mating-inducing condition. Data are presented as the mean ± SD of six independent experiments (two-tailed Student’s *t*-test). (D) Expression of Gis1-mCherry in cells cultured under filamentation-suppressing condition, GlcN-inducing condition or mating-inducing condition for 48 hrs. The boxplots represent the medians and interquartile ranges. For each condition, 120 cells were randomly selected and evaluated for fluorescence intensity. CV, coefficient of variation. Scale bar, 10 μm. (E) The expression of Gis1-mCherry in cells at pre-filamentation stage or undergoing filamentous growth under GlcN-inducing condition. The images of the fluorescence-labeled strains were taken 48 hrs and 7 days after incubation. For evaluation of fluorescence intensity, 120 yeast cells or hyphae were randomly selected and calculated for fluorescence intensity. Scale bar, 10 μm. (F) Diagram depicting experimental work flow designed to explore the correlation between Gis1 expression level and cell wall thickening ability under GlcN-inducing condition. (G) FACS-based cell sorting of strain harboring P_*GIS1*_*-GIS1-mCHERRY* based on Gis1-mCherry fluorescence level. Cells were cultured on YPGlcN medium for 48 hrs and cells were sorted by FACS in the mCherry low (10% of total population) and mCherry high (10% of total population) categories, respectively. (H) Normalized cell wall thicknesses of cell populations with high and low expression of Gis1-mCherry, sorted by flow cytometry. The boxplots represent the medians and interquartile ranges. To exclude the potential influence of cell size on cell wall thickness, normalized cell wall thicknesses (cell wall thickness/cell diameter) were calculated. For each cell population, 50 yeast cells were randomly selected and used to calculate the normalized cell wall thickness. Two-tailed Student’s *t*-test. (I) Cell wall morphology (left panel) and thickness evaluation (right panel) of wild-type and *gis1*Δ mutant strains under GlcN-inducing condition or mating-inducing condition. For each strain, 100 yeast cells were randomly selected and used to calculate the cell wall thickness. The boxplots represent the medians and interquartile ranges. Two-tailed Student’s *t*-test. Scale bar, 100 nm.

During cryptococcal cellular differentiation, highly dynamic expression has been detected in many genes encoding secretory/surface proteins, which harbor a signal peptide destined for the secretory pathway [20,35,36]. In particular, earlier studies indicated that some of these proteins exhibit specificity for enrichment in certain stages of differentiation and can be used as stage-specific marker proteins [20,35,36]. To explore the potential marker protein of the GlcN-induced cell wall synthetic response, we selected 10 predicted secretory/surface protein-coding genes, which belonged to the gene group 1 and showed high transcriptional induction upon GlcN stimulation but no evident increase in their expression in response to mating stimulation based on the RNA-seq data of GIF and sexual filamentation [20] (Fig 3A). The corresponding mutants of these genes were subjected to phenotypic assays for evaluating their abilities to undergo GIF. Among them, only the disruption of CNAG_05036 severely attenuated GIF (Fig 3B). We named this gene *GIS1* (GlcN-induced surface protein 1), which encodes a hypothetical protein with no known protein domain, as revealed by Pfam domain prediction. To examine the temporospatial expression of Gis1 during GIF and mating filamentation, we fused the fluorescence protein mCherry with Gis1 that is driven by the *GIS1*’s native promoter. In agreement with the qRT-PCR results showing that *GIS1* was specifically induced during GIF but not in cells grown on YPD agar or under mating-inducing condition (Fig 3C), abundant Gis1-mCherry was detected largely on the cell surface upon GlcN induction, while mating cells or cells from YPD agar expressed this protein very weakly (Fig 3D). Interestingly, the expression of Gis1-mCherry was highly induced at the early stage associated with the pre-filamentation cell wall response, and its expression declined when cells underwent filamentous growth, suggesting that Gis1 is pre-filamentation stage-enriched (Fig 3E). We noticed that during pre-filamentation phase, yeast cells were highly heterogeneous in terms of Gis1-mCherry abundance based on fluorescence intensity measurement (Fig 3D). To probe the correlation between Gis1 expression level and cell wall thickening ability in GlcN-stimulated cells, we performed FACS-based cell sorting to collect different cell subpopulations with high and low Gis1-mCherry abundance, respectively (Fig 3F and 3G). As shown in Fig 3H, cells expressing high level of Gis1 showed a significantly thicker cell wall than Gis1 low-expressed cells, suggesting that Gis1 might be crucially involved in the cell wall synthesis induced by GlcN (Fig 3H). In consistence with this idea, the *gis1*Δ cells showed a dramatically decreased cell wall thickness in the presence of GlcN but not under mating-inducing condition (Fig 3I). Considering that the expression of Gis1 is greatly related to and required for the GlcN-induced cell wall synthetic response (Fig 3H and 3I), it could be used as an indicator molecule of this commitment stage of GIF.

### Mpk1 MAPK cascade is the central signaling pathway that bridges the cell wall response and GIF

**Fig 4:**
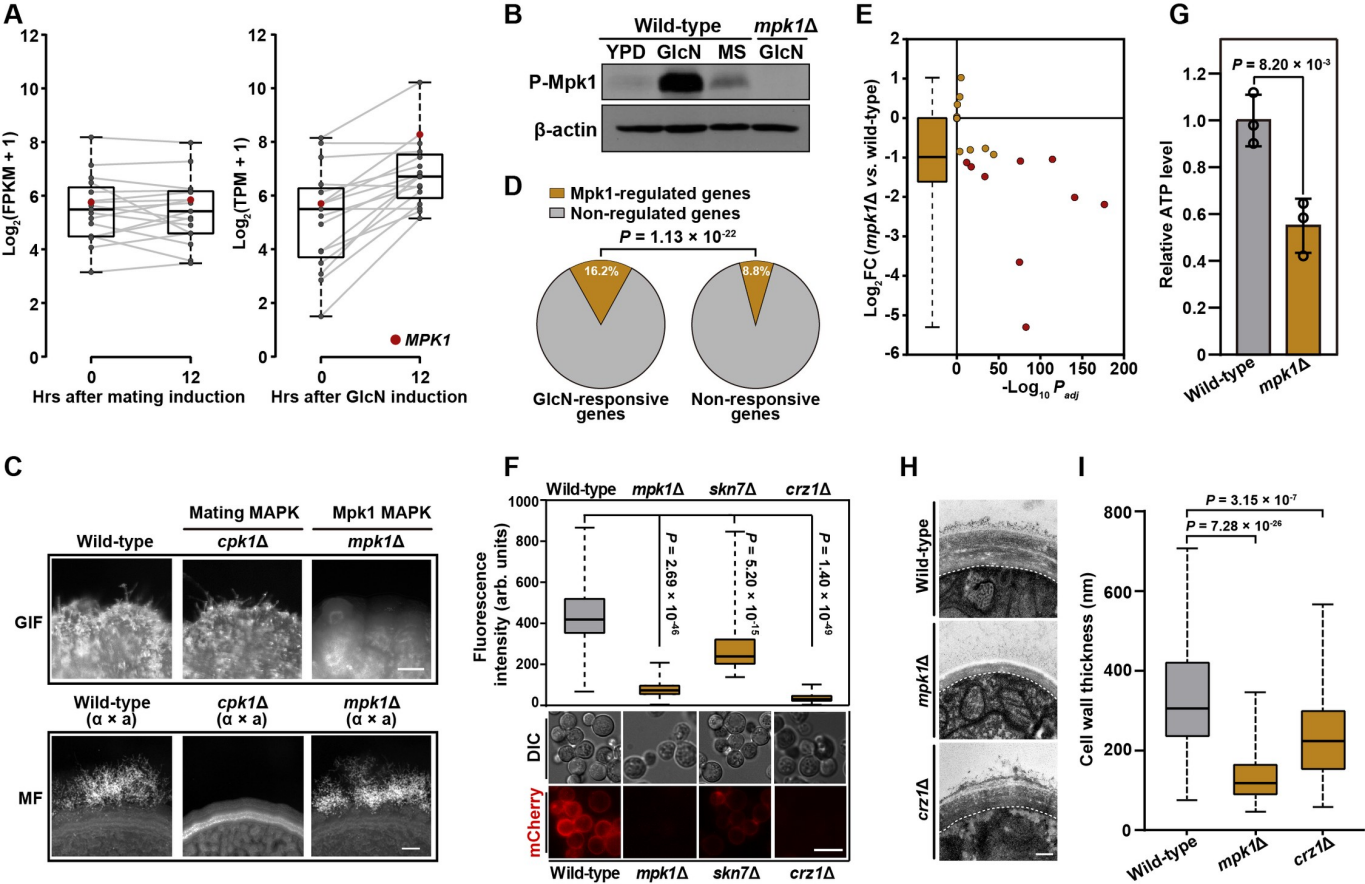
Mpk1 MAPK cascade is the central signaling pathway connecting the cell wall response and GIF. (A) The induction of genes belonging to the Mpk1 MAPK pathway in response to mating induction or GlcN stimulation. The boxplots represent the medians and interquartile ranges. The cryptococcal gene expression data under mating-inducing conditions were derived from our previous research [20]. The red circle represents *MPK1*. (B) Western blot analysis of phosphorylated Mpk1 in wild-type strain incubated on YPD, YPGlcN or MS medium for 6 hrs. The *mpk1*Δ mutant was used as negative control. The same blots were restriped and probed with β-actin antibody as the protein loading control. (C) Filamentation phenotypes of the wild-type, *cpk1*Δ and *mpk1*Δ mutant strains under GlcN-inducing (upper panel) and mating-inducing (bottom panel) conditions. MF, mating filamentation. Scale bars, 100 μm. (D) Percentage of Mpk1-regulated targets included in GlcN-responsive and non-responsive genes. Fisher’s exact test. (E) The effect of Mpk1 on the transcriptional response of GlcN-induced genes known or predicted to be involved in cell wall biogenesis or regulation [38,46,47]. The boxplots represent the medians and interquartile ranges. Red circle indicates gene whose transcription is significantly induced by Mpk1 during GIF (log_2_FC (*mpk1*Δ *vs*. wild-type) < -1, *P*_*adj*_ < 0.01). (F) Expression of Gis1-mCherry in cells of different strains cultured on GlcN-inducing media for 48 hrs. The images are representative of three independent experiments with similar results. For evaluation of fluorescence intensity, 100 yeast cells were randomly selected and calculated for fluorescence intensity. Scale bar, 10 μm. (G) Relative ATP levels of wild-type and *mpk1*Δ. The cellular ATP level was measured using cells cultured under GlcN-inducing condition for 48 hrs. Data are presented as the mean ± SD of three independent experiments, two-tailed Student’s *t*-test. (H) Cell wall morphology of the wild-type, *mpk1*Δ and *crz1*Δ mutant strains during GIF. The yeast cell wall structures of different strains were visualized by TEM after 48 hrs of incubation on YPGlcN agar. Scale bar, 200 nm. (I) Cell wall thickness of the wild-type, *mpk1*Δ and *crz1*Δ mutant strains during GIF. For each strain, 100 yeast cells were randomly selected and used to calculate the cell wall thickness. The boxplots represent the medians and interquartile ranges. Two-tailed Student’s *t*-test.

The Mpk1 MAPK pathway, also named the cell wall integrity (CWI) pathway, is central for mediating cell wall activities in fungi[37–41]. RNA-seq assays revealed that many genes from this cascade showed strong induction in response to GlcN stimulation (Fig 4A). In contrast, no evident induction for the known Mpk1 MAPK genes was observed during mating (Fig 4A). We also preformed Western blot experiments to examine the abundance of phosphorylated (active) form of Mpk1 (P-Mpk1), the key kinase of the CWI pathway, in response to GlcN or the mating induction. Unlike mating cells that only had very weak level of P-Mpk1, abundant P-Mpk1 was detected in cells cultured under GIF-inducing condition (Fig 4B). The transcriptional and Western blot evidence suggest that the Mpk1 MAPK pathway is strongly activated by GlcN. Furthermore, we showed that the absence of any constituents of the core MAPK cascade (Bck1-Mkk2-Mpk1) abolished GIF (Figs 4C and S4A); however, the ability to undergo mating filamentation was not adversely influenced in *mpk1*Δ according to bilateral mating assays (Fig 4C). We also constructed the *MPK1* deletion mutant in *C*. *gattii* to test whether Mpk1 is likewise required for GIF in this pathogen. As expected, deletion of *MPK1* prevented GIF based on phenotypic analysis (S3G Fig). These data suggest that the Mpk1-MAPK pathway governs GIF as a key signaling cascade in different *Cryptococcus* pathogens.

To determine the effect of the Mpk1 MAPK pathway on mediating the cell wall response to GlcN, we performed transcriptomic analysis targeting *mpk1*Δ in the presence of GlcN. The RNA-seq results indicated that 931 genes were differentially expressed in *mpk1*Δ compared with the wild-type strain (|log_2_(fold change)| > 1, *P*_*adj*_ < 0.01) (S3 Table). Among Mpk1-regulated genes, ~46.7% were GlcN-responsive genes, which displayed differential expression after the yeast cells were transferred onto the medium containing GlcN. These genes accounted for 16.2% of the total *C*. *neoformans* GlcN-responsive genes. By comparison, only 8.8% of the total none-responsive genes were controlled by Mpk1 (Fig 4D and S3 Table). These results suggest an important role of Mpk1 in mediating GlcN response. Of note, the increased expression of a majority of cell wall-related genes in response to GlcN induction were substantially weakened in the *mpk1*Δ cells based on comparative transcriptomic analysis (Fig 4E). These genes included the coding gene of Gis1 (S3 Table), the indicator molecule of the GlcN-induced cell wall synthetic response. Consistently, deletion of *MPK1* severely reduced the expression of mCherry tagged Gis1 under GIF-inducing condition (Fig 4F). In addition to activating cell wall biosynthetic genes, Mpk1 also promotes ATP production that is critical for the energy-consuming pre-filamentation cell wall synthesis induced by GlcN, as revealed by the observation that the cellular ATP level was markedly lower in the *mpk1*Δ mutant than that in wild-type during GIF (Fig 4G). Moreover, the *mpk1*Δ cells displayed a cell wall thickness almost comparable to that of the wild-type strain without GlcN induction (Figs 2H, 2I, 4H and 4I). Besides, the multilayer cell wall structure stimulated by GlcN in wild-type was undetectable in the *mpk1*Δ mutant during GIF (Fig 4H and 4I). These results support the core role of the Mpk1 MAPK pathway in directing the pre-filamentation cell wall synthetic response during GIF.

### Phosphoproteomics profiling systematically identified the regulatory network downstream of Mpk1 during GIF

**Fig 5:**
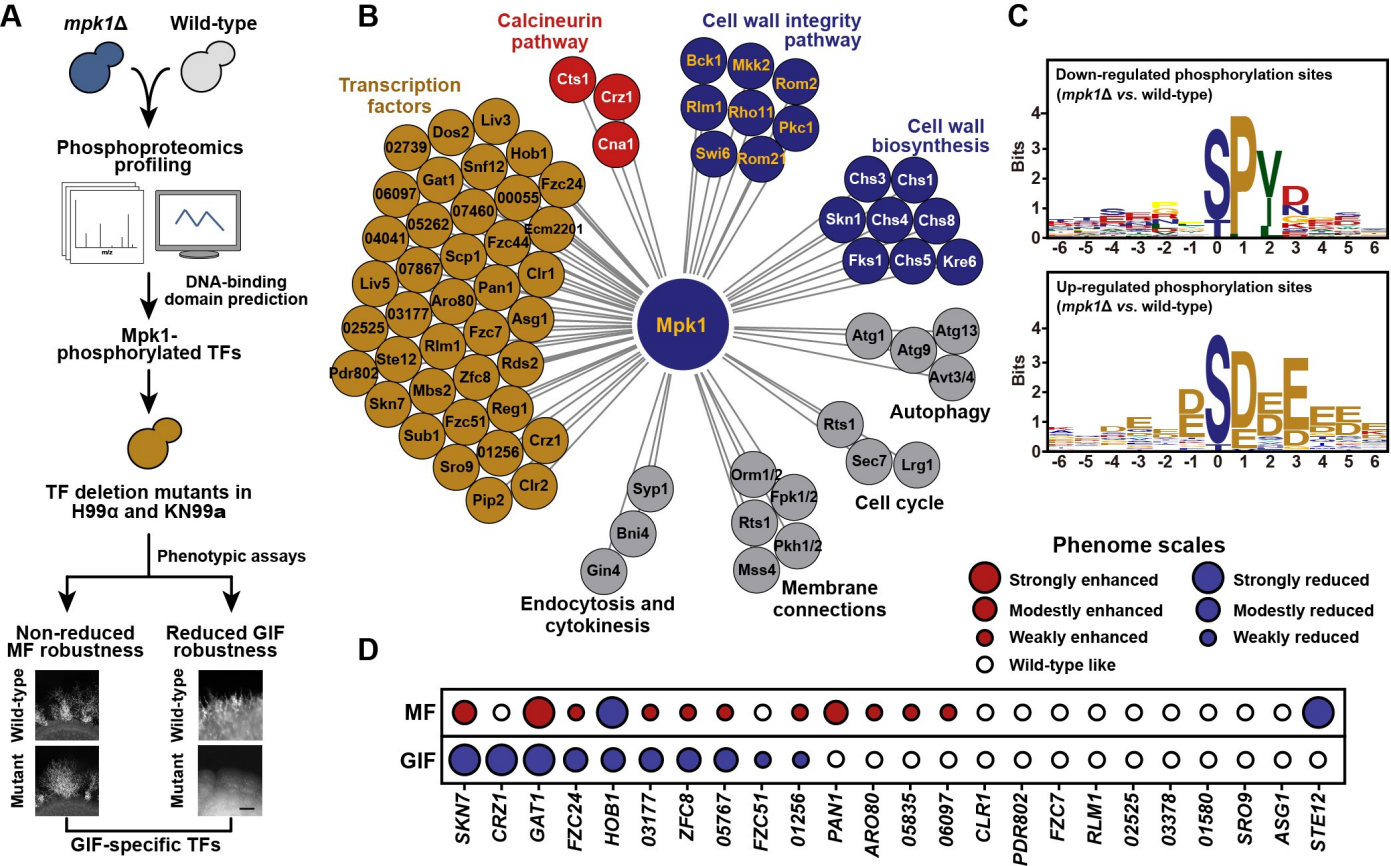
Quantitative phosphoproteomics reveals the phosphorylation motif and candidate substrate proteins for Mpk1. (A) Schematic diagram depicting the phosphoproteomics-based method combined with genetic/phenotypic analysis applied to identify the Mpk1-phosphorylated transcription factors, which underlie the regulatory specificity towards GIF. MF, mating filamentation. (B) Functional categorization of Mpk1 substrate proteins identified by phosphoproteomics analysis. CNAG_‘number’ shown in the “Transcription factors” part indicates the gene locus name used in Broad Institute *C*. *neoformans* H99 sequence annotation, and these locus names were used because they are previously uncharacterized genes. All proteins shown here are targets of Mpk1 that were identified based on phosphoproteomics analysis. (C) The enriched phosphorylation motifs predicted in upregulated (upper panel) or downregulated (bottom panel) phosphorylation events in response to the absence of Mpk1. (D) Semiquantitative phenotypic assays unveiled the contribution of the Mpk1-phosphorylated TFs to mating-induced filamentation or GIF. Red or blue circles indicate enhanced or reduced filamentation, respectively. Circle size represents the relative robustness of filamentation. CNAG_0‘number’ indicates the NCBI locus name of the previously uncharacterized gene.

In the model yeast *Saccharomyces cerevisiae*, Rlm1 is the major regulator downstream of the cell wall integrity pathway [42–44]. However, the absence of its ortholog in *C*. *neoformans* only led to a marginal reduction in GIF (if any) (S4B Fig), and *rlm1*Δ had no detectable effect on typical cell wall weakening phenotypes, such as hypersensitivity to Congo red (S4B Fig). This finding suggests that Rlm1 might not be the effector of Mpk1 MAPK signaling in the basidiomycete *C*. *neoformans*, which diverged from the ascomycete *S*. *cerevisiae* at least 500 million years ago [45]. To identify the important regulator(s) downstream of Mpk1 MAPK signaling that control the GlcN-induced cell wall synthetic response and dimorphism, we used phosphoproteomics profiling with three biological repeats to explore the targets phosphorylated by Mpk1 in response to GlcN (Fig 5A). Using this method, we identified a total of 12100 phosphosites on 2798 proteins at 6 hrs post GlcN induction in the wild-type H99 strain. We detected remarkable changes, including 1070 phosphorylation and 1272 de-phosphorylation events, for 1153 of these proteins when Mpk1 was absent (S4 Table). These potential Mpk1 targets contain many proteins responsible for cell wall biosynthesis or belong to the cell wall integrity pathway [38,46,47] (Fig 5B). In addition, we identified Mpk1 targets responsible for the calcineurin pathway, the autophagy, cell cycle, membrane connections, endocytosis and cytokinesis that were documented to be affected by Mpk1 in other fungal species [38,48–54] (Fig 5B). These results highlight the reliability of our phosphoproteomics experiments. Among the downregulated phosphorylation sites in response to Mpk1 depletion, the likelihood of a proline at the + 1 position relative to the S/T is over-represented and this pattern is consistent with the previously reported consensus motif recognized by MAP kinases [55] (Fig 5C). In particular, this motif was detected in many of the published cell wall biosynthetic proteins and the Mpk1 MAPK pathway components with downregulated phosphorylation sites [38,46,47] (S5 Table). In comparison, the consensus motif of Mpk1 was barely observed in the upregulated phosphorylation sites, which are anticipated to be attributed to indirect effect caused by the absence of Mpk1 (Fig 5C).

### The Mpk1 MAPK pathway governs the cell wall response and GIF through a coordinated regulatory network, with Skn7 and Crz1 being the core components

Multiple transcriptional factors (TFs) displayed a remarkably reduced phosphorylation in the absence of Mpk1 during GIF (Fig 5B). Of these TFs, twenty-four TFs contain the putative Mpk1 phosphorylation motif (Fig 5D and S6 Table). These TFs include Crz1, the calcineurin downstream zinc finger transcription factor [56]. Crz1 was shown to activate GIF but to be not engaged in mating filamentation [24,57]. However, unlike Crz1, the upstream calcineurin components, such as the catalytic subunit Cna1, are equally vital for GIF and mating filamentation [24,58]. This indicates existence of another signaling pathway that regulates the activity or expression of Crz1 for enabling the regulatory specificity of GIF. We showed that like the *mpk1*Δ mutant, the *crz1*Δ mutant similarly displayed impaired cell wall synthetic response to GlcN: it had significantly thinner cell wall and the considerably attenuated expression of Gis1-mCherry compared with wild-type after GlcN stimulation (Fig 4F, 4H and 4I). qRT-PCR analysis indicated that *CRZ1* and *MPK1* are reciprocally regulated in the presence of GlcN (S5A Fig), suggesting a cross-talk between Mpk1 and Crz1 during GIF. This idea was further verified by comparative transcriptomic analysis, which showed that more than 62% of the targets of Crz1 were also controlled by Mpk1 (S5B Fig and S3 Table). In addition, the regulatory pattern of cell wall-related genes by Mpk1 was highly similar to that controlled by Crz1 during GIF (S5C Fig, *r* = 0.90, *P* = 5.69 × 10^−12^, Pearson correlation analysis).

**Fig 6:**
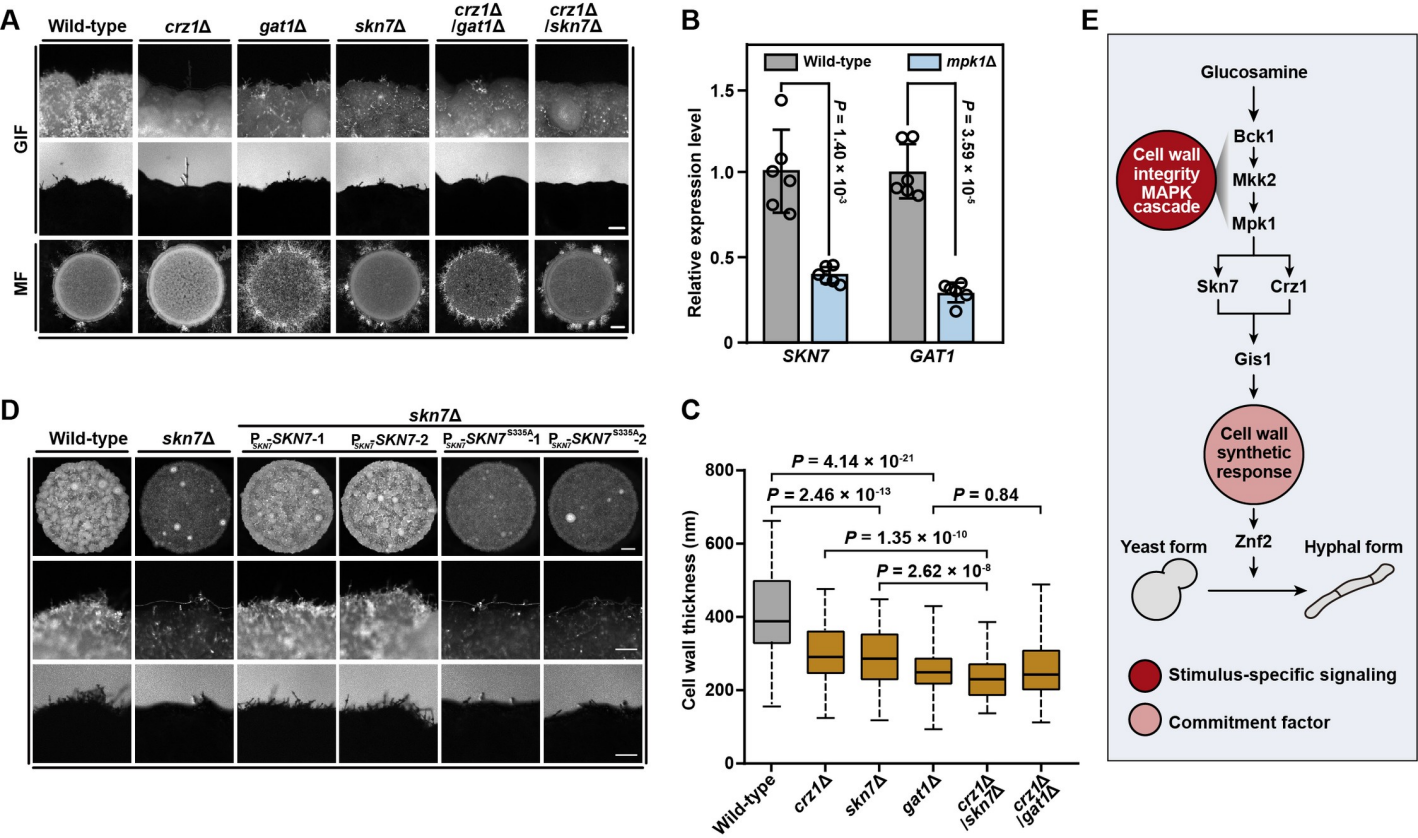
Mpk1 governs the cell wall response and GIF through Skn7 and Crz1. (A) Phenotypes of GIF and mating filamentation in various mutants. For GIF, cells of different strains were cultured on YPGlcN agar for 7 days. For mating filamentation (MF), equal numbers of α and **a** cells were cultured on MS agar for 14 days. Scale bars, 100 μm (middle panel), 1 mm (bottom panel). (B) qRT-PCR showing the changes in the mRNA levels of *SKN7* and *GAT1* in the absence of Mpk1. Data are presented as the mean ± SD of six independent experiments (two-tailed Student’s *t*-test). (C) Cell wall thickness of various mutants. For each strain, 100 yeast cells were randomly selected and used to calculate the cell wall thickness. The boxplots represent the medians and interquartile ranges. Two-tailed Student’s *t*-test. (D) Filamentation phenotypes of wild-type, *skn7*Δ and the complemented strains of *skn7*Δ expressing Skn7 or Skn7^S335A^ in response to GlcN. Cells of different strains were cultured on YPGlcN agar for 7 days. For *skn7*Δ/P_*SKN7*_-*SKN7* or *skn7*Δ/P_*SKN7*_-*SKN7*^S335A^, two independent transformants were applied for phenotypic assessment. Scale bar, 100 μm. (E) Proposed model of the regulatory hierarchy controlling GlcN-induced filamentation in *Cryptococcus neoformans*.

The phenotypic assays for GIF robustness revealed that disruption of *CRZ1*, while causing a dramatically attenuated filamentation as reported previously [24], cannot completely prevent it, which can otherwise be achieved by deleting *MPK1* (S5D Fig). Additionally, in comparison to the absence of Crz1, the depletion of Mpk1 caused a more severe defect in cell wall growth stimulated by GlcN, as indicated by TEM evaluation (Fig 4H and 4I). These phenotypic dissimilarities suggest the presence of other TF(s) downstream of Mpk1 that function in concert with Crz1 during GIF. To identify such TF(s), we obtained 48 α and **a** congenic deletion strains for 24 Mpk1-phosphorylated TFs either from available *Cryptococcus* gene deletion libraries [26] (16 α mutants) or through *de novo* gene deletion (8 α mutants and 24 **a** mutants) (Fig 5D and S7 Table). Semiquantitative phenotypic analysis was applied to these mutants to evaluate their impact on GIF and sexual filamentation generated via bilateral crosses (Fig 5D). We showed that 10 out of 24 TFs (~42%) were involved in GIF activation (Fig 5D). Moreover, 90% of these GIF regulators were dispensable for the induction of mating dimorphism, suggesting their contribution to the stimulatory specificity towards GIF (Fig 5D). Two regulators (the response regulator Skn7 and the GATA family TF Gat1) appear to be the most important GIF-specific activators in addition to Crz1: the absence of either of them strongly inhibited GIF and the cell wall response to GlcN, but caused enhanced mating filamentation (Figs 5D and 6A). Transcriptional evaluation based on qRT-PCR indicated that disruption of *MPK1* significantly reduced the mRNA levels of *SKN7* and *GAT1* during GIF (Fig 6B), suggesting that they are important transcription factors downstream of Mpk1. The phenotypic analysis indicated that *skn7*Δ/*crz1*Δ but not *gat1*Δ/*crz1*Δ completely abolished the GlcN-induced yeast-to-hypha transition, as observed in the *mpk1*Δ mutant (Figs 4C and 6A). Conversely, double deletion of *SKN7* and *CRZ1* did not inhibit mating filamentation (Fig 6A). Furthermore, the cells lacking both Skn7 and Crz1 displayed a significantly thinner cell wall than either of the cells with a single deletion after GlcN stimulation (Fig 6C). In comparison, *gat1*Δ/*crz1*Δ displayed a similar level of cell wall thickness to *gat1*Δ (Fig 6C). These findings indicate that Skn7 functions together with Crz1 to ensure the stimulatory specificity towards GIF, which is a cell wall response-dependent process. Furthermore, similar to *crz1*Δ, *skn7*Δ showed an attenuated expression of Gis1, furthering confirming their important roles in the Gis1-dependent cell wall response (Fig 4F).

The phosphoproteomic analysis showed that in the absence of Mpk1, Skn7 showed decreased phosphorylation at Ser335 adjacent to the HSF DNA binding domain (S6A and S6B Fig). To test whether the phosphorylation of Skn7 by Mpk1 is required for its stimulatory activity towards GIF, the *skn7*Δ mutant was introduced with the gene encoding the mutated Skn7, in which the potential phosphorylation site was substituted with alanine (S6C Fig). At least five independent transformants were evaluated for GIF robustness with two of them shown in Fig 6D. We showed that the filamentous robustness of all transformants tested was similar to that of the *skn7*Δ mutant, suggesting that the substitution did not restore the defect of GIF (Fig 6D). In contrast, introduction of the intact version of *SKN7* led to filamentous robustness comparable to that of the wild-type strain upon GlcN induction (Fig 6D).

## Discussion

Many fungi that are known to cause invasive infections in humans can undergo transition between yeast and hypha in response to different external cues [16,59–63]. The examples are thermally dimorphic fungal pathogens, in which temperature is the predominant environmental cue that drives cellular differentiation [16,63,64]. These pathogens normally live as filaments at mild temperatures during saprophytic life cycles and as pathogenic yeast or yeast-like cells at 37°C in the host [16,63,64]. In addition to thermal cues, many other external stimuli have been documented to induce or modulate dimorphic transition in various human fungal pathogens [65–68]. For instance, GlcNAc, a monomer of chitin, has been reported to facilitate yeast-to-filament conversion in thermally dimorphic fungi and *Candida* species that belong to the Ascomycota phylum [67,69–72]. Unlike these ascomycetous pathogens, GlcNAc does not have a stimulatory effect on filamentation in the basidiomycete *C*. *neoformans* [24], which diverged from ascomycetes more than 900 million years ago [73]. Instead, filamentation in this basidiomycetous pathogen can otherwise be activated by GlcN [24], the deacetylated form of GlcNAc. Time-series RNA-seq analysis together with TEM evaluation indicated that GlcN induces a global cell wall response in *C*. *neoformans*, which is characterized by synchronized induction of biosynthetic genes for different cell wall components (Figs 2G and S3A). This transcriptional response results in substantially enhanced cell wall synthesis before GIF in *C*. *neoformans* (Fig 2H and 2I). This suggests that GlcN, as a chitosan-derived monosaccharide, play a stimulatory role in cell wall synthesis in *C*. *neoformans*. Perturbation of the cell wall synthesis response by CR abolished GlcN-induced dimorphism, suggesting that this cellular response represents a key commitment factor underlying GIF (Fig 2F).

We showed that the Mpk1 MAPK pathway is the central cascade that mediates the cell wall response in *C*. *neoformans* (Fig 4D, 4E and 4H). In *S*. *cerevisiae*, Rlm1 is the key output regulator of the Slt2/Mpk1 MAPK pathway [42,43]. However, its ortholog in *C*. *neoformans* does not importantly contribute to GIF (S4B Fig). This finding suggests that Rlm1 seems unlikely to be the core regulator of cell wall integrity signaling for GIF activation in *C*. *neoformans*. Our evidence together with previous research showed that Crz1 displayed similar functions as Mpk1 in the activation of cell wall response and GIF [24,56,57] (Figs 4H, 4I and S5D). Comparative transcriptomic analysis also revealed a highly shared gene set regulated by Crz1 and Mpk1 during GIF, suggesting an important regulatory relationship between them (S5B Fig and S3 Table). Our phosphoproteomic assessment indicated that Mpk1 likely phosphorylates Crz1 in a direct manner (Fig 5B). However, Crz1 may not be the effector of cell wall integrity pathway in *C*. *neoformans* since the phosphorylation of Crz1 by Mpk1 is not important for its function in GIF activation, as proven by alanine replacement mutagenesis (S7 Fig). The transcriptional evidence indicated that in the presence of GlcN, *CRZ1* and *MPK1* were positively regulated by each other in transcriptional level, suggesting that they may undergo indirect crosstalk during GIF (S5A Fig).

We further explored that Skn7, as a key target of Mpk1, functions with Crz1 in the activation of the cell wall response (Fig 6E). Unlike Crz1, the phosphorylation of Skn7 by Mpk1 is critical for its activity, suggesting that Skn7 is an important target of Mpk1 in GIF activation (Fig 6D). Skn7 is a response regulator that is highly conserved among fungi [74]. In budding yeast, Skn7 together with a hybrid sensor histidine kinase (HK) and phosphotransfer (HPt) protein constitute a phosphorelay system, known as the “two component system” [75,76]. In this system, Skn7 receives the phosphorylation signal from HPt, which is phosphorylated by HK in response to extracellular stimuli [75,76]. *C*. *neoformans* has seven HKs (Tco1-7) and one HPt protein (Ypd1), whose coding gene is essential in this fungus [77,78]. We observed substantially weakened GIF in the mutant strain devoid of the HK protein Tco2 (S2A and S2B Fig). This result suggests that Tco2-directed two component system and the Mpk1 MAPK pathway may cooperate to activate GIF through Skn7.

Notably, the co-absence of Skn7 and Crz1 resulted in a thinner cell wall than that of either of the single deletions but this change was not as severe as that observed in *mpk1*Δ (Figs 4H, 4I and 6C). This finding suggests that additional factors downstream of Mpk1 are involved in the cell wall response. This idea is consistent with the result showing that in addition to Skn7 and Crz1, seven TFs are also specific for GIF activation (Fig 5D). Thus, it is conceivable that Mpk1 directs the cell wall response and GIF through an integrated regulatory network formed by multiple TFs, including Skn7 and Crz1. In *C*. *neoformans*, Mpk1 MAPK signaling is the core pathway for cell wall adaptation to chemical and physical challenges from hosts and the environment [79–81]. In this regard, further functional dissection of the regulatory network downstream of Mpk1 revealed by us will help comprehensively elucidate the pleotropic roles of Mpk1 MAPK signaling in the infection and biology of *C*. *neoformans*.

## Materials and methods

### Strains and growth conditions

The strains used in this study are listed in S7 Table. The kinase and transcription factor deletion sets made in the H99 background were obtained from the Fungal Genetics Stock Center (FGSC, http://www.fgsc.net/crypto/crypto.htm). Strains were routinely grown on YPD (1% yeast extract, 2% Bacto Peptone, 2% dextrose, and 2% Bacto Agar) medium at 30°C. GlcN-induced filamentation assays were performed on YPGlcN (1% yeast extract, 2% Bacto Peptone, 2% GlcN, and 2% Bacto Agar) medium as described previously [24]. Mating assays were performed on V8 agar (0.5 g/liter KH_2_PO_4_, 4% Bacto Agar and 5% V8 juice, pH = 5.0) or Murashige and Skoog (MS) medium minus sucrose (Sigma-Aldrich) at 25°C in the dark. For CR tolerance, overnight cultures of different strains were washed and diluted to an *A*_600_ = 1.0 and five-fold serial dilutions were generated. Three microliters of each dilution was dropped on a YPD plate and YPD plate supplemented with 1% CR.

### Filamentation assay

GlcN-induced filamentation assays were performed as described previously with modifications [24]. Briefly, the strains were grown on YPD plates for 24 hours. The cells were collected and washed twice with ddH_2_O and diluted to a final *A*_600_ = 1.0. Three microliters of the cells was then dropped onto YPGlcN medium and incubated at 30°C for 7 days. For mating-induced filamentation, the cells of H99 **a**-derived and α-derived strains were collected and washed twice with ddH_2_O and diluted to a final *A*_600_ of 1.0. Equal volumes of **a** and α cells were mixed and 3 microliters of the mixture was dropped onto MS medium and incubated at 25°C in the dark for 2 weeks. For analysis of the effect of perturbation of the cell wall response to GlcN on GIF, YPGlcN and MS medium was supplemented with 0.0025% Calcofluor white or 0.0025% Congo red. Notably, cell wall inhibitors at these concentrations or even higher concentrations (up to 0.1%) cannot affect cryptococcal growth.

### Gene knockout and complementation

Targeted gene deletion was performed as previously described [10]. Briefly, an ~1 kb fragment flanking the gene coding region was amplified and fused with the NEO (neomycin) resistance cassette. The deletion construct was introduced into the relevant recipient strain by the TRACE method as previously described [82]. Gene replacement was genetically and transcriptionally confirmed by diagnostic PCR and qRT-PCR, respectively. The primers used for the generation of the mutants are listed in S7 Table.

### Measurements of cellular ATP

Cells of different strains were incubated on MS medium or YPGlcN medium or YPGlcN medium supplemented with 1 mM NaAsO_2_ for 48 hrs. The cells were subsequently harvested and washed three times with ice-cold sterilized water at 4°C. After lyophilization, cells were lysed using glass beads and ATP levels were quantified using a BacTiter-Glo Microbial Cell Viability Assay Kit (Promega, G8231) and a luminometer (Glomax-20/20, Promega Biosystems, USA) following the manufacturer’s protocol.

### Protein extraction and Western blot analysis

Strains were incubated for 48 hrs on YPGlcN or MS plate. The cells were harvested and frozen in liquid nitrogen before protein extraction. After lyophilization, the cells were lysed using glass beads, and the cell pellets were resuspended in 800 μL lysis buffer (10 mM Tris-HCl, pH 7.5, 150 mM NaCl, 0.5 mM EDTA, 1% SDS, 1% Triton X-100, 1% deoxycholate, 5% glycerol, 2 mM Na_3_VO_4_, 10 mM Na_4_P_2_O_7_, 25 mM NaF, and 1 × protease inhibitor cocktail) and incubated for 10 min on ice. Lysates were centrifuged at 12,000 rpm at 4°C for 10 min, and the supernatants were collected. A Coomassie (Bradford) protein assay kit (Beyotime, no. P0006C) was used to determine the protein concentration. Equal amounts of protein were loaded onto Tris-glycine gel (10%) and transferred to polyvinylidene difluoride membrane (Millipore). To examine phosphorylated Mpk1, a 1:2, 500 dilution of phospho-p44/42 MAPK (Thr202/Tyr204) rabbit polyclonal antibody (Cell Signaling Technology, no. 4370) was used. Subsequently, the blot was stripped, and a rabbit β-actin monoclonal antibody (GenScript, no. A00702) was used as a loading control.

### Secretory/cell surface protein prediction

The signal peptide and subcellular localization of the proteins were predicted based on publicly available SignalP (http://www.cbs.dtu.dk/services/SignalP/) and WoLF PSORT (https://wolfpsort.hgc.jp/) programs, respectively.

### Microscopy and fluorescence

Strains harboring P_*GIS1*_-*GIS1*-*mCHERRY* were grown on YPGlcN agar at 30°C for 48 hrs or 168 hrs to examine the subcellular localization of Gis1. Calcofluor White (1 mg/mL) was used for cell wall staining before microscopic examination. Images were acquired using a Zeiss Axioplan 2 imaging system with the AxioCam MRm camera (Carl Zeiss Microscopy). The fluorescence intensity of Gis1-mCherry in yeast cells or hyphae was analyzed using software Zen 2011 (Carl Zeiss Microscopy).

### Site-directed mutagenesis

PCR-based site-directed mutagenesis was carried out to mutate Ser335 to alanine in *SKN7* and Ser569 to alanine in *CRZ1* using the QuikChange site-directed mutagenesis kit (Agilent Technologies). Mutations in the constructs were confirmed by Sanger sequencing. Constructs expressing wild-type protein (Skn7 or Crz1) or mutated protein (Skn7^S335A^ or Crz1^Ser569A^) under the control of their native promoters were subsequently introduced to the Safe Heaven (SH2) site [83] in relevant gene deletion strains (*crz1*Δ or *skn7*Δ) by the TRACE method [82]. The primers used for mutagenesis are listed in S8 Table.

### Cell-cell fusion assay

Cell-cell fusion assays were performed as previously described with modifications [21]. H99α and KN99**a** strains harboring neomycin or nourseothricin resistance genes were grown on YPD plates for 2 days. The cells were collected and washed twice with ddH_2_O and diluted to a final *A*_600_ of 1.0. Equal numbers of α and **a** cells were mixed, and 10 microliters of the mixture was spotted onto V8 agar and YPGlcN agar. After incubation for 15 hrs at 25°C (V8 agar) or 60 hrs at 30°C (YPGlcN agar), the cells were then collected, and plated in serial dilutions on both YPD media and YPD media supplemented with both G418 and nourseothricin. The cells were incubated at 30°C for four days. CFUs (colony-forming units) were counted, and the cell-cell fusion frequency was calculated based on the average number of double drug-resistant CFUs/total CFUs.

### Transmission electron microscopy

Transmission electron microscopy was performed at the Beijing Regional Center of Life Science Instrument, Chinese Academy of Science. Strains were incubated on YPD, MS medium, YPGlcN or YPGlcN supplemented with 1% CR or 1 mM NaAsO_2_ at 30°C for 48 hrs. The cells were subsequently harvested and washed with sterilized water and subsequently fixed with 2.5% glutaraldehyde in 0.1 M phosphate buffer (pH 7.2) overnight at 4°C. After the samples were rinsed with ddH_2_O three times, they were dehydrated stepwise with methanol. The dehydrated pellets were then embedded in Epon 812 resin for sectioning. The ultrathin sections were subsequently stained with 2% uranium acetate and images were observed under a Tecnai Spirit 120 kV transmission electron microscope (FEI, USA).

### Flow cytometry

Strain harboring P_*GIS1*_-*GIS1*-*mCHERRY* was incubated on YPGlcN medium at 30°C for 48 hrs. The cells were harvested and washed with sterilized water and subsequently fixed with 2.5% glutaraldehyde in 0.1 M phosphate buffer (pH 7.2) overnight at 4°C. The samples were then washed with PBS, strained through a 40 μm mesh filter and diluted to 10^7^ cells/mL. 10^6^ cells were sorted by FACS in the mCherry low (10% of total population) and mCherry high (10% of total population) categories, respectively. FACS experiments were performed using BD FACSAriaIII and data were analyzed using FlowJo software (TreeStar).

### RNA purification and quantitative RT-PCR analysis

Total RNA extraction and quantitative real-time PCR (qRT-PCR) were carried out as previously described [84]. The cells were ground to a fine powder in liquid nitrogen, and total RNA was then extracted and purified using an Ultrapure RNA Kit (Kangweishiji, CW0581S) according to the manufacturer’s instructions. Reverse transcription was performed using the FastQuant RT Kit (Tiangen KR106-02) following the manufacturer’s instructions. The relative expression level of selected genes was determined using Power SYBR qPCR premix reagents (KAPA) in a CFX96 Touch Real-time PCR detection system (Bio-Rad). The relative transcript levels of the genes were calculated as a fold change and normalized to the housekeeping gene *TEF1* using the comparative *C*_*t*_ method. The primers used for qRT-PCR are listed in S8 Table.

### RNA-seq and data analysis

Strains were cultured in YPD liquid medium at 30°C for 12 hrs. The cells were collected, washed twice with ddH_2_O, and diluted to *A*_600_ = 5.0. The diluted cells were spotted on YPGlcN medium and incubated at 30°C for 0, 6, 12, 24, 36, 48 and 60 hrs. Total RNA was subsequently extracted from the cells collected at different time points. The RNA concentration of each sample was assessed using a Qubit RNA Assay Kit on a Qubit 2.0 Fluorometer (Life Technologies, CA, USA). The RNA integrity was evaluated using the RNA Nano 6000 Assay Kit with a Bioanalyzer 2100 system (Agilent Technologies, CA, USA). The transcriptome libraries were generated using the VAHTS mRNA-seq v2 Library Prep Kit (Vazyme Biotech Co., Ltd, Nanjing, China) following the manufacturer’s protocols. Transcriptome library sequencing was performed by Annoroad Gene Technology Co., Ltd (Beijing, China). The samples were clustered using VAHRS RNA adapters set1/set2 and then sequenced on an Illumina platform. Initial quality control of the sequenced clean data was performed using FastQC v0.11.5 software. After the initial quality control, ~2 GB of clean data of each sample (representing over 100 × coverage) was mapped to the genome sequence of H99 using STAR_2.6.0c. The gene-expression level was measured in TPM to determine unigenes using StringTie v1.3.3. All unigenes were subsequently aligned against the well-annotated genome of H99. The differentially expressed genes were assessed using the DEseq2 v1.16.1 Bioconductor package and defined based on the fold change criterion (|log_2_(fold change)| > 1, *P*_*adj*_ < 0.01). In all RNA-seq assays performed in this study, more than two biological replicates were included. For temporal expression signature analysis, GlcN-regulated genes (fold change ≥ 2 and R-squared ≥ 0.75) were identified based on the generalized additive model, and their normalized counts across different time courses were used to group genes according to the DP_GP_cluster method using default parameters. The method uses the Dirichlet process Gaussian process mixture model (DPGP) to analyze time-series RNA-seq data. The Dirichlet process (DPs) is used to determine the number of clusters and the Gaussian process (GPs) models the time-dependency and trajectory of gene expression [85]. Gene lists were uploaded into the PANTHER database (http://pantherdb.org/) to obtain the enriched GO terms.

### Phosphoproteomics

Phosphoproteomics sample processing, TMT labeling, phosphopeptide enrichment and LC-MS/MS analysis are fully detailed as follows:

- Protein extraction. Cells of the wild-type and *mpk1*Δ mutant strains were collected after incubation on YPGlcN medium for 6 hrs at 30°C. Cell pellets were washed three times with ice-cold water and ground to a fine powder in liquid nitrogen. The cell powder was collected and added to 4 mL of lysis buffer (8 M urea, 1% protease inhibitor cocktail), followed by sonication on ice using a high intensity ultrasonic processor (Scientz). Finally, the supernatant was collected after centrifugation at 12,000 g at 4°C for 10 min.
- Trypsin digestion. Protein in the supernatant was reduced with 5 mM DTT at 56°C for 30 min and alkylated with 11 mM iodoacetamide at room temperature in the darkness for 15 min. The protein sample was diluted by adding 100 mM triethylammonium bicarbonate (TEAB) to a urea concentration less than 2 M and then digested overnight with a 1:50 trypsin-to-total protein mass ratio.
- TMT labeling. The resulting peptides were desalted by a Strata X C18 SPE column (Phenomenex) and dried by vacuum. Peptides were reconstituted in 0.5 M TEAB and processed using a TMT kit (Thermo Fisher Scientific).
- HPLC fractionation. The TMT-labeled sample mixture was solubilized and fractionated into fractions using a Thermo Betasil C18 column (5 μm particles, 10 mm ID, 250 mm length). Different HPLC fractions were separated with a gradient of 8% to 32% acetonitrile (pH = 9.0) and vacuum-dried.
- Affinity enrichment. Peptide mixtures were incubated with IMAC microsphere suspensions with vibration in loading buffer (50% acetonitrile/6% trifluoroacetic acid). Phosphopeptides enriched by IMAC microspheres were collected by centrifugation and the supernatant was removed. The IMAC microspheres were sequentially washed with 50% acetonitrile/6% trifluoroacetic acid and 30% acetonitrile/0.1% trifluoroacetic acid. Enriched phosphopeptides from the IMAC microspheres were then collected and lyophilized for LC-MS/MS analysis.
- LC-MS/MS analysis. Peptides were dissolved in solvent A (0.1% formic acid) and loaded onto a reversed-phase analytical column (15 cm length, 75 μm ID). The gradient was comprised of an increase from 4% to 22% solvent B (0.1% formic acid in 90% acetonitrile) in 38 min and 22% to 32% in 14 min, climbing to 80% in 4 min and then holding at 80% for 3 min, all at a constant flow rate of 450 nL/min on an EASY-nLC 1000 UPLC system. The peptides were then subjected to the NSI source followed by tandem mass spectrometry (MS/MS) in Q Exactive Plus (Thermo Fisher Scientific) coupled online with the UPLC system. The electrospray voltage was set at 2.0 kV. The m/z scan range was set between 350 and 1600 for full scan, and intact peptides were detected in the Orbitrap at a resolution of 60,000. Peptides were then selected for MS/MS using the NCE setting of 28, and the fragments were detected in the Orbitrap at a resolution of 17,500. A data-dependent procedure that alternated between one MS scan followed by 20 MS/MS scans with 15.0 s dynamic exclusion. Automatic gain control (AGC) was set at 1E5. The fixed first mass was set as 100 m/z.

### Phosphoproteomic data analysis

MS/MS data were processed using MaxQuant v.1.5.2.8 against UniProt C*ryptococcus neoformans var*. *grubii* (https://www.uniprot.org/proteomes/?query=taxonomy:178876) database (n = 7430 entries). Trypsin/P was designated as a cleavage enzyme, allowing up to 4 missing cleavages. The mass tolerance of the precursor ion was set to 20 ppm in the first search, 5 ppm in the main search, and 0.02 Da for the fragment ion. The carbamoylmethyl group on Cys was designated as a fixed modification, and the phosphorylation on Ser/Thr/Tyr and oxidation on Met were designated as variable modifications. The FDR was adjusted to < 1%, and the minimum score for the modified peptides was set to > 40. Quantitative values for each condition were obtained from three biological replicates.

### Motif analysis

Soft MeMe (version 5.1.1) was used to analyze the model of sequences. The maximum number of motifs was set to 20. The motif width was set to 13 (6 amino acids upstream and downstream of the site). Other parameters used default settings.

### Statistical analyses

Statistical analyses were performed using R version 3.4.2. We used a two-tailed Student’s *t*-test to compare the cell wall thickness and transcript levels between two groups. Fisher’s exact test was utilized to evaluate the significance between two sets of genes. A two-sided *P* < 0.05 was considered significant, and *P* < 0.001 was considered very significant. The data are shown as the mean ± SD from three or more independent experiments. Statistical tests and experiment numbers are detailed in the figure legends.

## Supporting information

S1 Fig GlcN inhibits the mating efficiency of *C*. *neoformans*. (A) Cells were spotted on YPGlcN medium at initial density of OD_600_ = 1.0 and incubated at 30°C. Cellular morphology was observed at the indicated time points. Scale bar, 10 μm. (B) Equal numbers of α and **a** cells were cocultured on V8 agar or YPGlcN. The cell-cell fusion frequency was calculated as described in the Materials and Methods. ND, not detected. Data shown are from two independent experiments.
(TIF)

S2 Fig GIF robustness of *Cryptococcus* kinase deletion mutants. (A) Low-density cells of different strains were plated onto GlcN medium and cultured for 3 days to form isolated mini-colonies. The mini-colonies exhibited heterogeneity in filamentation and the strength of GIF was reflected by the filamentous incidence in mini-colonies. The kinase deletion mutants constructed by Yong-Sun Bahn’s group were obtained from the Fungal Genetics Stock Center. Scale bar, 100 μm. (B) Phenotype scores are indicated in distinct colors based on semiquantitative phenotypic evaluation of 129 kinase mutant strains. Transcriptional dynamics for each kinase were determined according to time-series RNA sequencing data targeting GIF. (C) A receiver operating characteristic (ROC) curve was used to evaluate the accuracy of the approach employed to evaluate phenotypic traits related to GIF robustness in 129 kinase mutants. AUC, area under the ROC curve.
(TIF)

S3 Fig GlcN stimulates global cell wall biosynthesis in a Znf2-independent manner. (A) Transcriptional dynamics for the cell wall-related genes revealed by time-series RNA-seq analysis during GIF. (B) Cell wall morphology (left panel) and the number of cell wall layers (right panel) of H99 yeast cells cultured on media containing glucose or GlcN. Cells cultured on medium containing GlcN exhibit multilayers of cell wall, but only one layer of cell wall could be observed when the cells were cultured on medium containing glucose. The boxplots represent the medians and interquartile ranges. For each condition, 100 yeast cells were randomly selected and used to count the layers of cell wall. Glc, Glucose. Scale bar, 500 nm. (C) Relative ATP levels of H99 cells incubated under GIF-inducing condition or mating-inducing condition for 48 hrs in the absence or presence of 1 mM NaAsO_2_. Data are presented as the mean ± SD of four independent experiments, two-tailed Student’s *t*-test. (D) RNA-seq-guided transcriptional analysis of the GlcN-induced cell wall-related genes in the *znf*2Δ mutant compared with wild-type in response to GlcN stimulation. RNA-seq experiments were performed using cells of the wild-type and *znf*2Δ mutant cultured on YPGlcN for 12 hrs. The boxplots represent the medians and interquartile ranges. (E) *C*. *gattii* cells were cultured on media containing glucose (Glc), glucosamine (GlcN) or glucosamine plus Congo red (GlcN + CR) for 48 hrs and the cell wall morphology of the yeast cells were visualized by TEM (transmission electronic microscope). Scale bar, 200 nm. (F) Quantification of cell wall thickness (bottom panel) of *C*. *gattii* strain cultured on media containing glucose (Glc), glucosamine (GlcN) or glucosamine plus Congo red (GlcN + CR). The boxplots represent the medians and interquartile ranges. For each condition, 100 yeast cells were randomly selected and used to calculate the cell wall thickness. Glc, Glucose. Two-tailed Student’s *t*-test. (G) Filamentation phenotypes of *C*. *gattii* wild-type and *mpk1*Δ cultured on YPGlcN agar with or without CR. Scale bars, 1 mm (upper panel), 200 μm (bottom panel).
(TIF)

S4 Fig Rlm1 is not the effector of the Mpk1 MAPK pathway during GIF. (A) GIF phenotypes of the Mpk1 MAPK cascade kinase deletion mutants. The absence of any constituent of the core MAPK cascade (Bck1-Mkk2-Mpk1) can completely abolish GIF. Scale bar, 200 μm. (B) Phenotypes of CR tolerance (upper panel) and GIF (bottom panel) of wild-type, *mpk1*Δ and *rlm1*Δ. Two independent transformants of the *RLM1* deletion mutant were applied for phenotypic assessment. Scale bar, 200 μm.
(TIF)

S5 Fig Transcriptomic analysis revealed crosstalk between Mpk1 and Crz1 during GIF. (A) qRT-PCR showing the mRNA levels of *MPK1* and *CRZ1* in different strains. RNA was extracted from the strains cultured on YPGlcN agar for 12 hrs. Data are presented as the mean ± SD of six independent experiments (two-tailed Student’s *t*-test). (B) Venn diagram analysis indicating the overlap between the Mpk1-regulated genes and the Crz1-regulated genes in response to GlcN stimulation. Fisher’s exact test. (C) RNA-seq-guided transcriptional analysis of cell wall-related genes in the *mpk1*Δ and *crz1*Δ mutant strains compared with the wild-type in response to GlcN stimulation. Spearman’s rank correlation analysis. The gray dashed line indicates log_2_(fold change) = -1. (D) Filamentation phenotypes of the wild-type, *mpk1*Δ and *crz1*Δ mutant strains in response to GlcN stimulation. Cells of different strains were spotted on YPGlcN medium and incubated at 30°C for 7 days. Scale bar, 100 μm.
(TIF)

S6 Fig Ser335 in Skn7 displayed a significantly reduced phosphorylation level in the absence of Mpk1 revealed by phosphoproteomics assessment. (A) MS/MS spectrum of the phosphoptide KTNNQEGDENpS^335^PR. The relative intensities of the TMT reporter ions show the changes in their phosphorylation levels. The insert is a zoom of spectrum to show the relative abundance of reporter ions from three biological repeats. (B) Schematic diagram of the functional domain contained in Skn7. (C) DNA sequence chromatograms of *SKN7* and *SKN7*^S335A^. The red arrow represents mutation site.
(TIF)

S7 Fig In Crz1, the phosphorylation of Ser569 by Mpk1 was not necessary for its function in GIF activation. (A) MS/MS spectrum of the phosphopeptide SDSIIPpS^569^PTADSFDR. The relative intensities of the TMT reporter ions showed the changes in their phosphorylation levels. The insert is a magnification of the spectrum to show the relative abundance of reporter ions from three biological replicates. (B) Filamentation phenotypes of wild-type, *crz1*Δ, and the complemented strains of *crz1*Δ expressing Crz1 or Crz1^S569A^ in response to GlcN. Cells of different strains were cultured on YPGlcN agar for 7 days. Scale bar, 100 μm.
(TIF)

S1 Table Time-series RNA-seq dataset for GIF development. (XLSX)

S2 Table Time-series transcriptional data (TPM values) of 129 kinase genes during GIF. (XLSX)

S3 Table Genes differentially expressed in *znf2*Δ, *mpk1*Δ and *crz1*Δ mutants during GIF. (XLSX)

S4 Table Mpk1-regulated phosphosites identified by phosphoproteomics. (XLSX)

S5 Table Phosphopetides from cell wall-related proteins identified in wild-type and *mpk1*Δ by phosphoproteomics. (XLSX)

S6 Table Phosphopeptides of TFs harboring putative Mpk1 phosphorylation motif identified by phosphoproteomics. (XLSX)

S7 Table Strains used in this study. (XLSX)

S8 Table Primers used in this study. (XLSX)
