# Supplementary figures and images for: A unique cell wall synthetic response evoked by glucosamine determines pathogenicity-associated fungal cellular differentiation
Source: PLoS Genet. 2021 Oct 8;17(10):e1009817. doi: 10.1371/journal.pgen.1009817 (PMC8500725; doi:10.1371/journal.pgen.1009817)

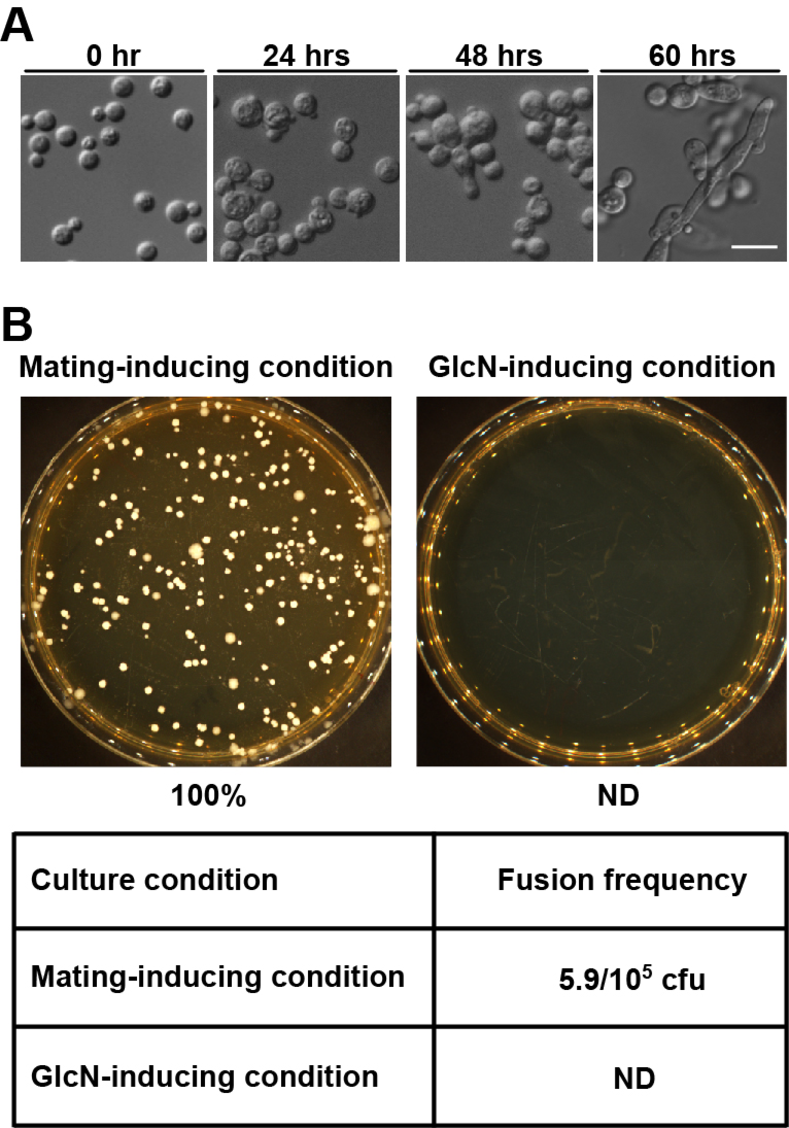

Supplement: S1 Fig — (A) Cells were spotted on YPGlcN medium at initial density of OD600 = 1.0 and incubated at 30°C. Cellular morphology was observed at the indicated time points. Scale bar, 10 μm. (B) Equal numbers of α and a cells were cocultured on V8 agar or YPGlcN. The cell-cell fusion frequency was calculated as described in the Materials and Methods. ND, not detected. Data shown are from two independent experiments. (TIF) [file pgen.1009817.s001.tif]

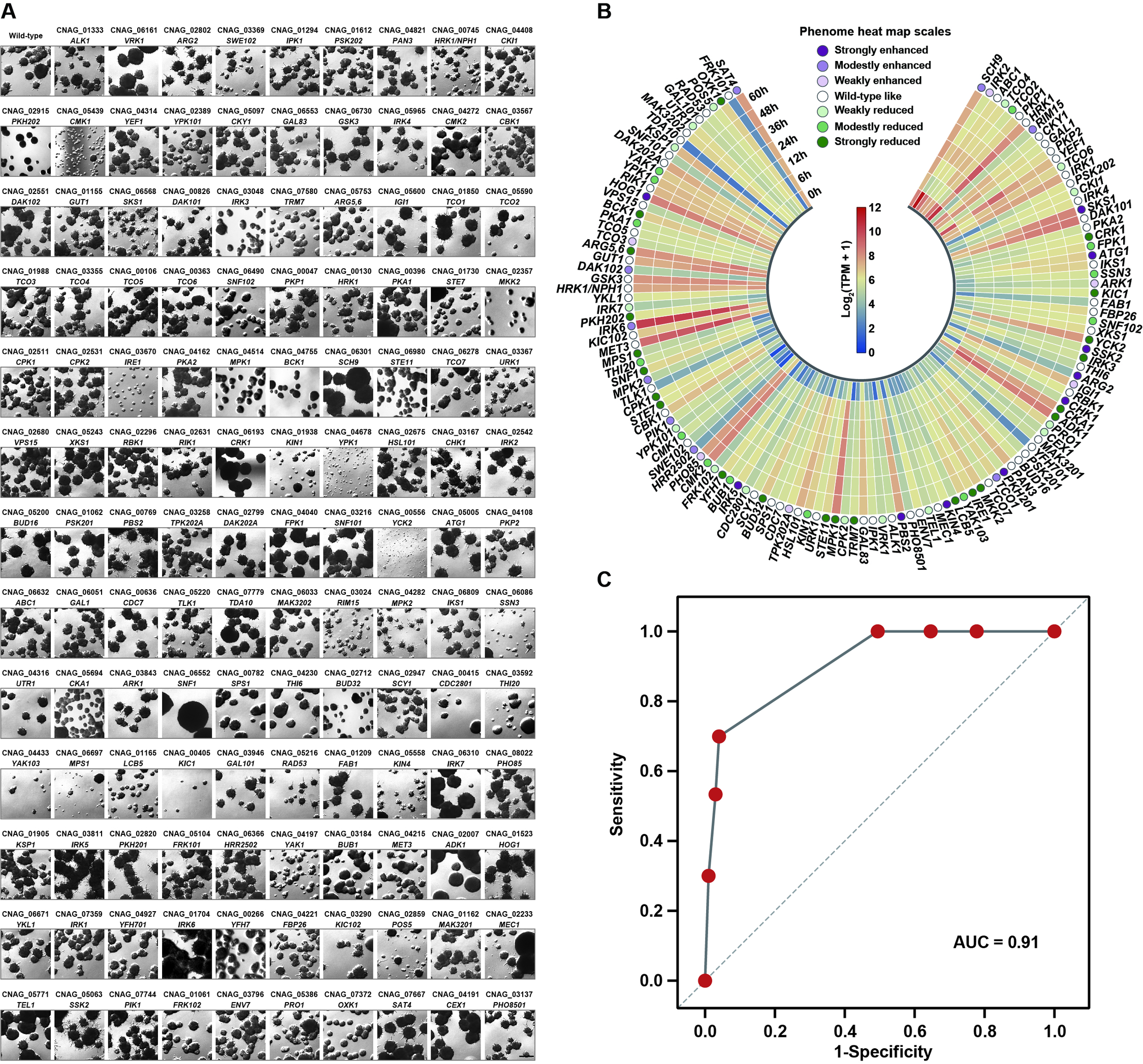

Supplement: S2 Fig — (A) Low-density cells of different strains were plated onto GlcN medium and cultured for 3 days to form isolated mini-colonies. The mini-colonies exhibited heterogeneity in filamentation and the strength of GIF was reflected by the filamentous incidence in mini-colonies. The kinase deletion mutants constructed by Yong-Sun Bahn’s group were obtained from the Fungal Genetics Stock Center. Scale bar, 100 μm. (B) Phenotype scores are indicated in distinct colors based on semiquantitative phenotypic evaluation of 129 kinase mutant strains. Transcriptional dynamics for each kinase were determined according to time-series RNA sequencing data targeting GIF. (C) A receiver operating characteristic (ROC) curve was used to evaluate the accuracy of the approach employed to evaluate phenotypic traits related to GIF robustness in 129 kinase mutants. AUC, area under the ROC curve. (TIF) [file pgen.1009817.s002.tif]

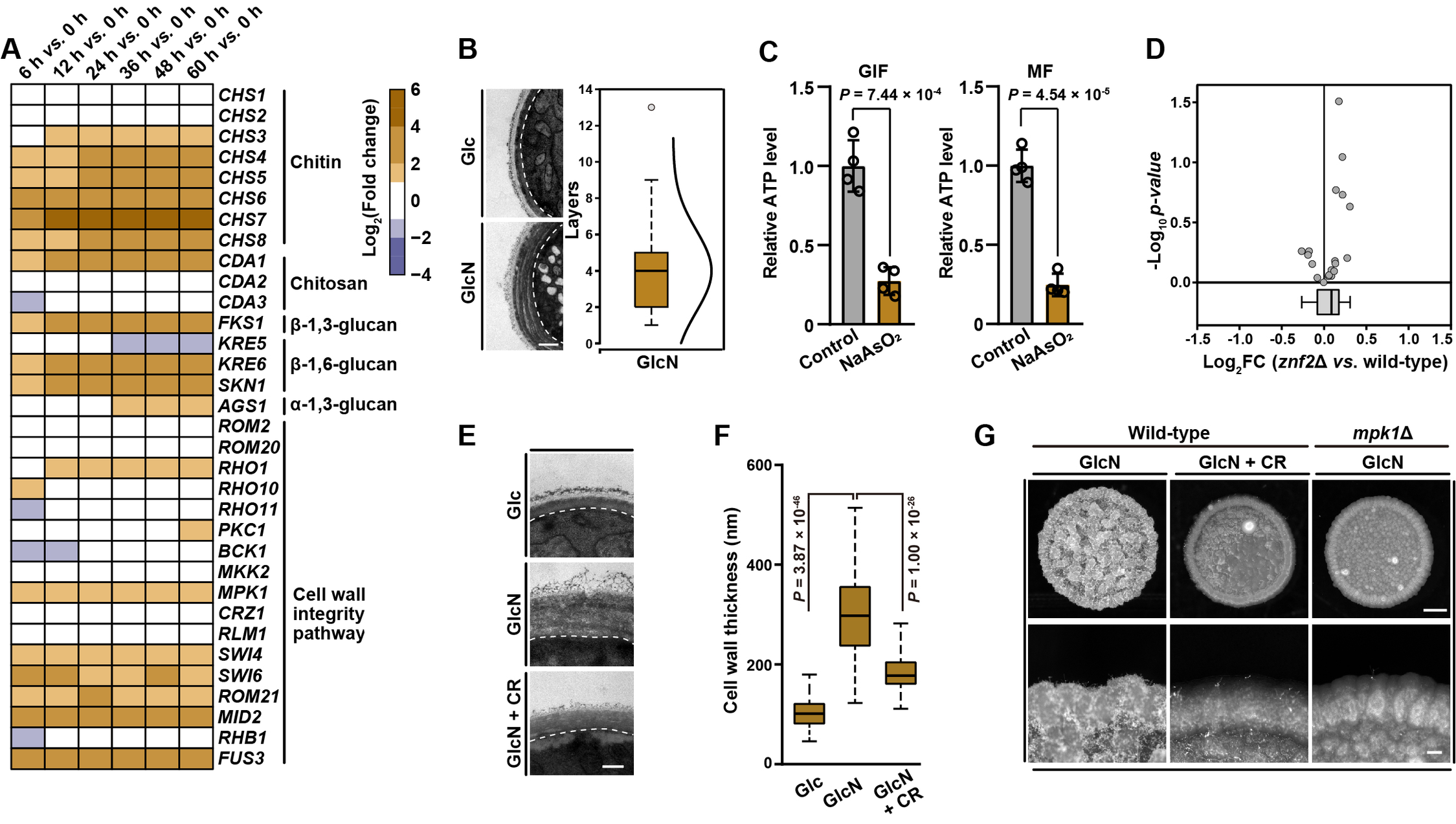

Supplement: S3 Fig — (A) Transcriptional dynamics for the cell wall-related genes revealed by time-series RNA-seq analysis during GIF. (B) Cell wall morphology (left panel) and the number of cell wall layers (right panel) of H99 yeast cells cultured on media containing glucose or GlcN. Cells cultured on medium containing GlcN exhibit multilayers of cell wall, but only one layer of cell wall could be observed when the cells were cultured on medium containing glucose. The boxplots represent the medians and interquartile ranges. For each condition, 100 yeast cells were randomly selected and used to count the layers of cell wall. Glc, Glucose. Scale bar, 500 nm. (C) Relative ATP levels of H99 cells incubated under GIF-inducing condition or mating-inducing condition for 48 hrs in the absence or presence of 1 mM NaAsO2. Data are presented as the mean ± SD of four independent experiments, two-tailed Student’s t-test. (D) RNA-seq-guided transcriptional analysis of the GlcN-induced cell wall-related genes in the znf2Δ mutant compared with wild-type in response to GlcN stimulation. RNA-seq experiments were performed using cells of the wild-type and znf2Δ mutant cultured on YPGlcN for 12 hrs. The boxplots represent the medians and interquartile ranges. (E) C. gattii cells were cultured on media containing glucose (Glc), glucosamine (GlcN) or glucosamine plus Congo red (GlcN + CR) for 48 hrs and the cell wall morphology of the yeast cells were visualized by TEM (transmission electronic microscope). Scale bar, 200 nm. (F) Quantification of cell wall thickness (bottom panel) of C. gattii strain cultured on media containing glucose (Glc), glucosamine (GlcN) or glucosamine plus Congo red (GlcN + CR). The boxplots represent the medians and interquartile ranges. For each condition, 100 yeast cells were randomly selected and used to calculate the cell wall thickness. Glc, Glucose. Two-tailed Student’s t-test. (G) Filamentation phenotypes of C. gattii wild-type and mpk1Δ cultured on YPGlcN agar with or wit [file pgen.1009817.s003.tif]

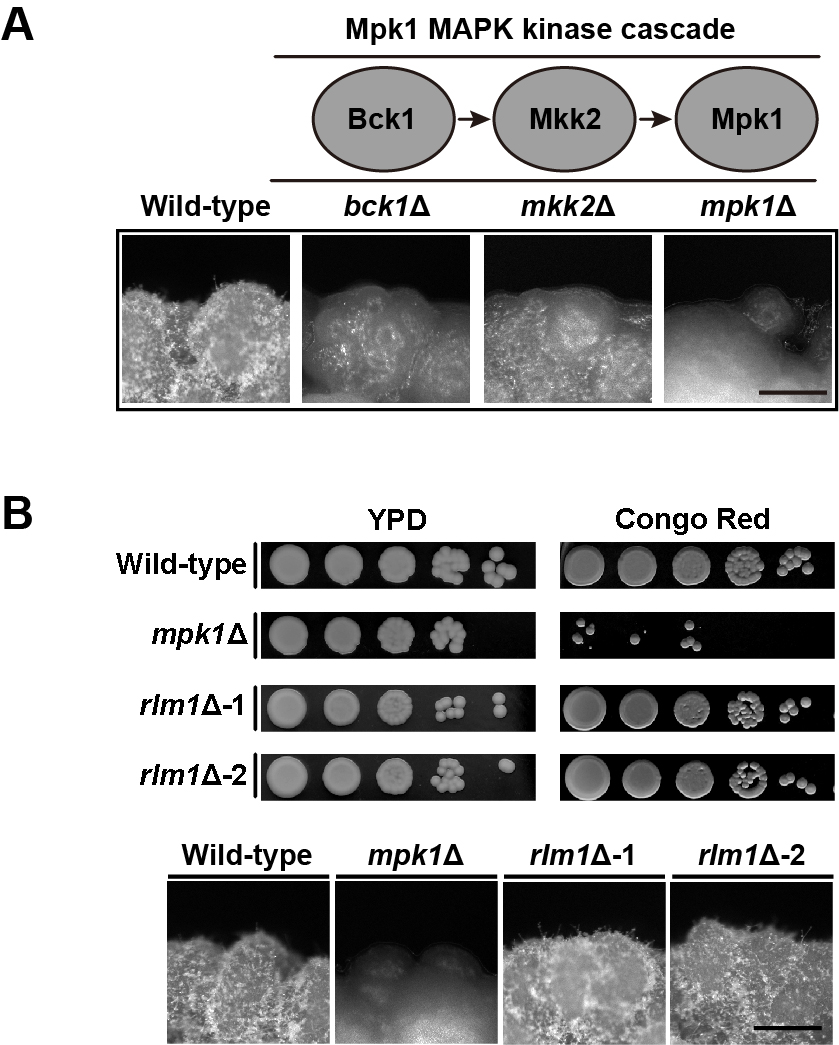

Supplement: S4 Fig — (A) GIF phenotypes of the Mpk1 MAPK cascade kinase deletion mutants. The absence of any constituent of the core MAPK cascade (Bck1-Mkk2-Mpk1) can completely abolish GIF. Scale bar, 200 μm. (B) Phenotypes of CR tolerance (upper panel) and GIF (bottom panel) of wild-type, mpk1Δ and rlm1Δ. Two independent transformants of the RLM1 deletion mutant were applied for phenotypic assessment. Scale bar, 200 μm. (TIF) [file pgen.1009817.s004.tif]

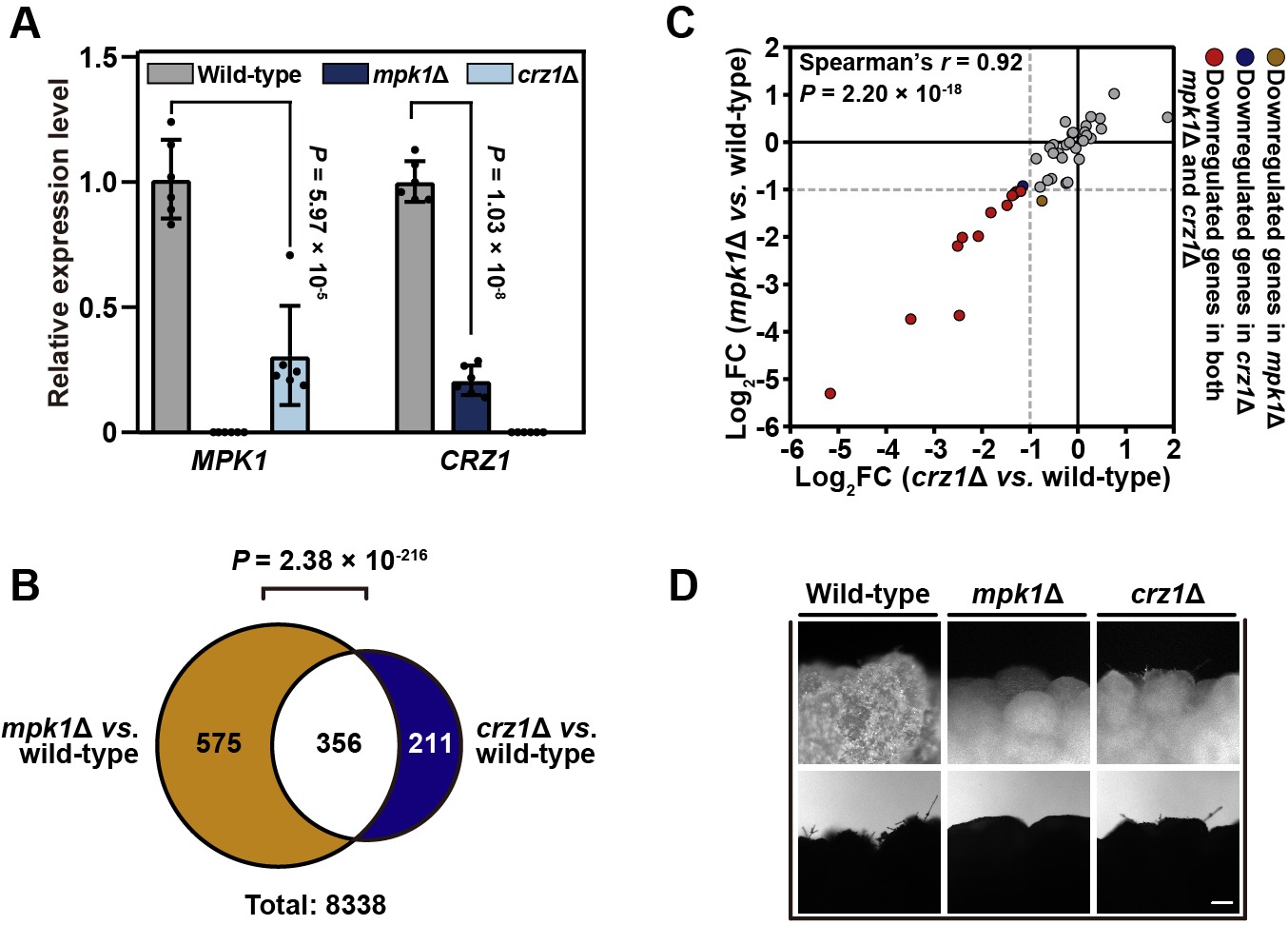

Supplement: S5 Fig — (A) qRT-PCR showing the mRNA levels of MPK1 and CRZ1 in different strains. RNA was extracted from the strains cultured on YPGlcN agar for 12 hrs. Data are presented as the mean ± SD of six independent experiments (two-tailed Student’s t-test). (B) Venn diagram analysis indicating the overlap between the Mpk1-regulated genes and the Crz1-regulated genes in response to GlcN stimulation. Fisher’s exact test. (C) RNA-seq-guided transcriptional analysis of cell wall-related genes in the mpk1Δ and crz1Δ mutant strains compared with the wild-type in response to GlcN stimulation. Spearman’s rank correlation analysis. The gray dashed line indicates log2(fold change) = -1. (D) Filamentation phenotypes of the wild-type, mpk1Δ and crz1Δ mutant strains in response to GlcN stimulation. Cells of different strains were spotted on YPGlcN medium and incubated at 30°C for 7 days. Scale bar, 100 μm. (TIF) [file pgen.1009817.s005.tif]

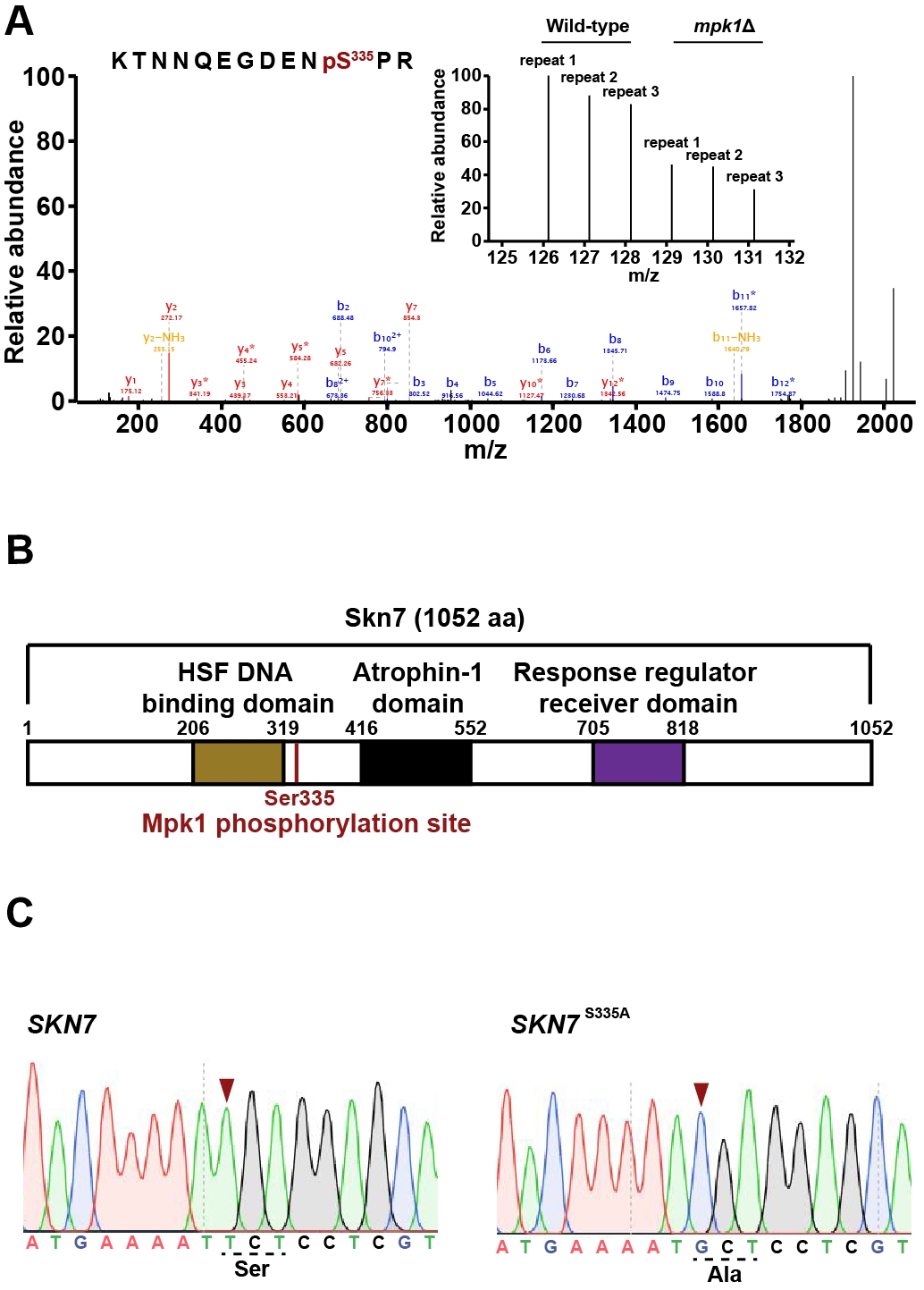

Supplement: S6 Fig — (A) MS/MS spectrum of the phosphoptide KTNNQEGDENpS335PR. The relative intensities of the TMT reporter ions show the changes in their phosphorylation levels. The insert is a zoom of spectrum to show the relative abundance of reporter ions from three biological repeats. (B) Schematic diagram of the functional domain contained in Skn7. (C) DNA sequence chromatograms of SKN7 and SKN7S335A. The red arrow represents mutation site. (TIF) [file pgen.1009817.s006.tif]

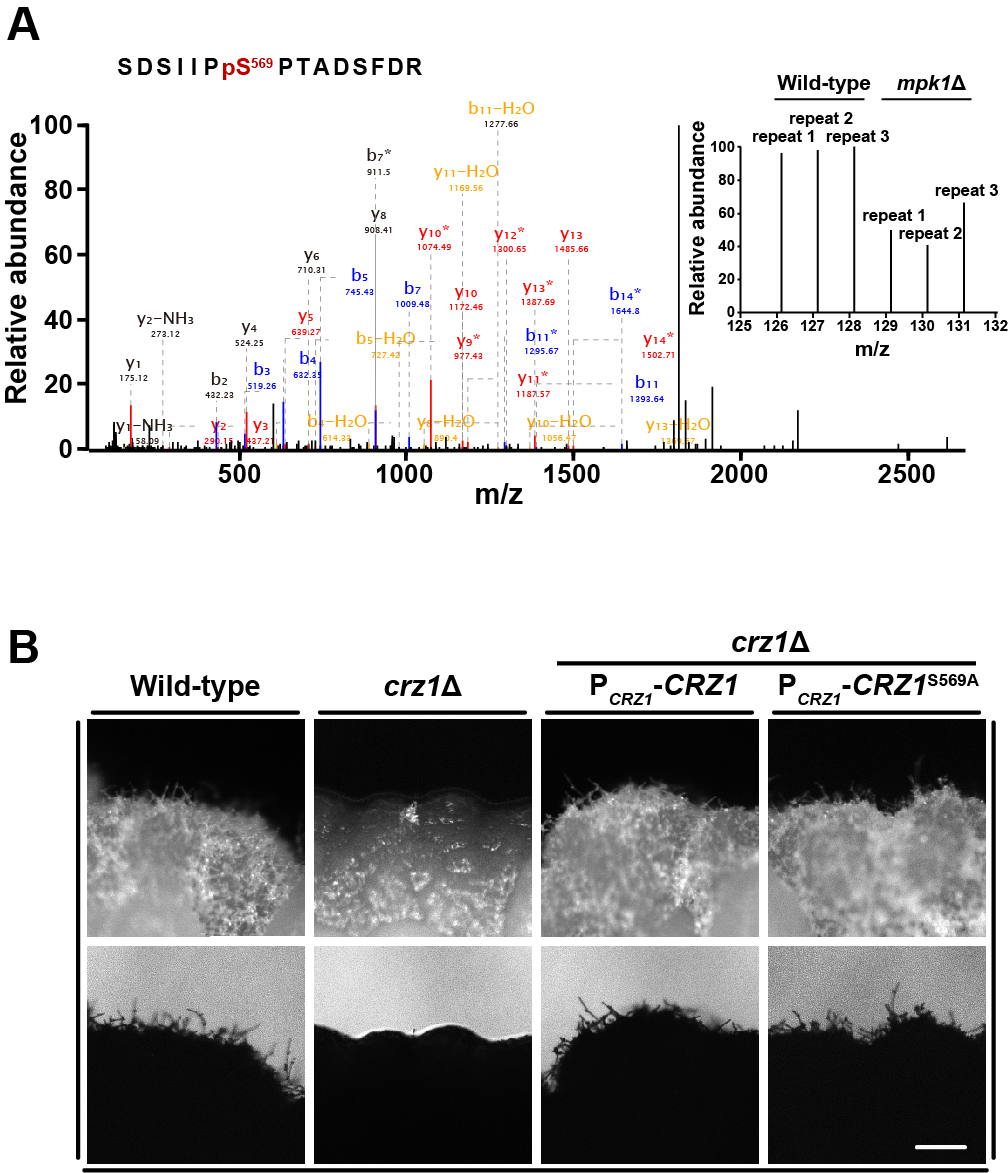

Supplement: S7 Fig — (A) MS/MS spectrum of the phosphopeptide SDSIIPpS569PTADSFDR. The relative intensities of the TMT reporter ions showed the changes in their phosphorylation levels. The insert is a magnification of the spectrum to show the relative abundance of reporter ions from three biological replicates. (B) Filamentation phenotypes of wild-type, crz1Δ, and the complemented strains of crz1Δ expressing Crz1 or Crz1S569A in response to GlcN. Cells of different strains were cultured on YPGlcN agar for 7 days. Scale bar, 100 μm. (TIF) [file pgen.1009817.s007.tif]
